# EGFR Signal-Network Reconstruction Demonstrates Metabolic Crosstalk in EMT

**DOI:** 10.1371/journal.pcbi.1004924

**Published:** 2016-06-02

**Authors:** Kumari Sonal Choudhary, Neha Rohatgi, Skarphedinn Halldorsson, Eirikur Briem, Thorarinn Gudjonsson, Steinn Gudmundsson, Ottar Rolfsson

**Affiliations:** 1 Center for Systems Biology, University of Iceland, Reykjavik, Iceland; 2 Biomedical Center, University of Iceland, Reykjavik, Iceland; 3 Stem Cell Research Unit, Department of Anatomy, School of Health Sciences, University of Iceland, Reykjavík, Iceland; 4 Department of Laboratory Hematology, Landspitali-University Hospital, Reykjavik, Iceland; University of Virginia, UNITED STATES

## Abstract

Epithelial to mesenchymal transition (EMT) is an important event during development and cancer metastasis. There is limited understanding of the metabolic alterations that give rise to and take place during EMT. Dysregulation of signalling pathways that impact metabolism, including epidermal growth factor receptor (EGFR), are however a hallmark of EMT and metastasis. In this study, we report the investigation into EGFR signalling and metabolic crosstalk of EMT through constraint-based modelling and analysis of the breast epithelial EMT cell model D492 and its mesenchymal counterpart D492M. We built an EGFR signalling network for EMT based on stoichiometric coefficients and constrained the network with gene expression data to build epithelial (EGFR_E) and mesenchymal (EGFR_M) networks. Metabolic alterations arising from differential expression of EGFR genes was derived from a literature review of AKT regulated metabolic genes. Signaling flux differences between EGFR_E and EGFR_M models subsequently allowed metabolism in D492 and D492M cells to be assessed. Higher flux within AKT pathway in the D492 cells compared to D492M suggested higher glycolytic activity in D492 that we confirmed experimentally through measurements of glucose uptake and lactate secretion rates. The signaling genes from the AKT, RAS/MAPK and CaM pathways were predicted to revert D492M to D492 phenotype. Follow-up analysis of EGFR signaling metabolic crosstalk in three additional breast epithelial cell lines highlighted variability in *in vitro* cell models of EMT. This study shows that the metabolic phenotype may be predicted by *in silico* analyses of gene expression data of EGFR signaling genes, but this phenomenon is cell-specific and does not follow a simple trend.

## Author Summary

The epidermal growth factor receptor (EGFR) signaling cascade is one of the key signaling pathways that are involved in the induction of Epithelial Mesenchymal Transition (EMT) and tumor metastasis. These signaling cascades often affect metabolic fate in tumor cells and control their progression. Here we demonstrate a method to build a mathematical model of the EGFR signaling cascade and use it to study signaling in EMT and how signaling affects metabolism. The model was used to obtain a list of potential signaling and metabolic targets of EMT. These targets may aid in the understanding of the molecular mechanisms that underlie EMT and metastasis. Our results further highlight the heterogeneity of cell models used to study EMT and support the idea of cell specific anti-cancer interventions.

## Introduction

Epithelial to mesenchymal transition (EMT) is a developmental process where polarized epithelial cells transition to an invasive mesenchymal-like phenotype through molecular reprogramming that leads to degradation of the extra-cellular matrix (ECM) and the loss of cell polarity. Following recruitment to specific sites at distant locations within the developing embryo, the mesenchymal cells may revert back to the epithelial phenotype by a process known as mesenchymal to epithelial transition (MET), thereby seeding new epithelial tissues [1]. Although EMT is fundamental for several developmental processes and wound healing, dysregulation of EMT may cause cancer cells to initiate metastasis and form secondary tumors at distant sites [1–3].

**Fig 1:**
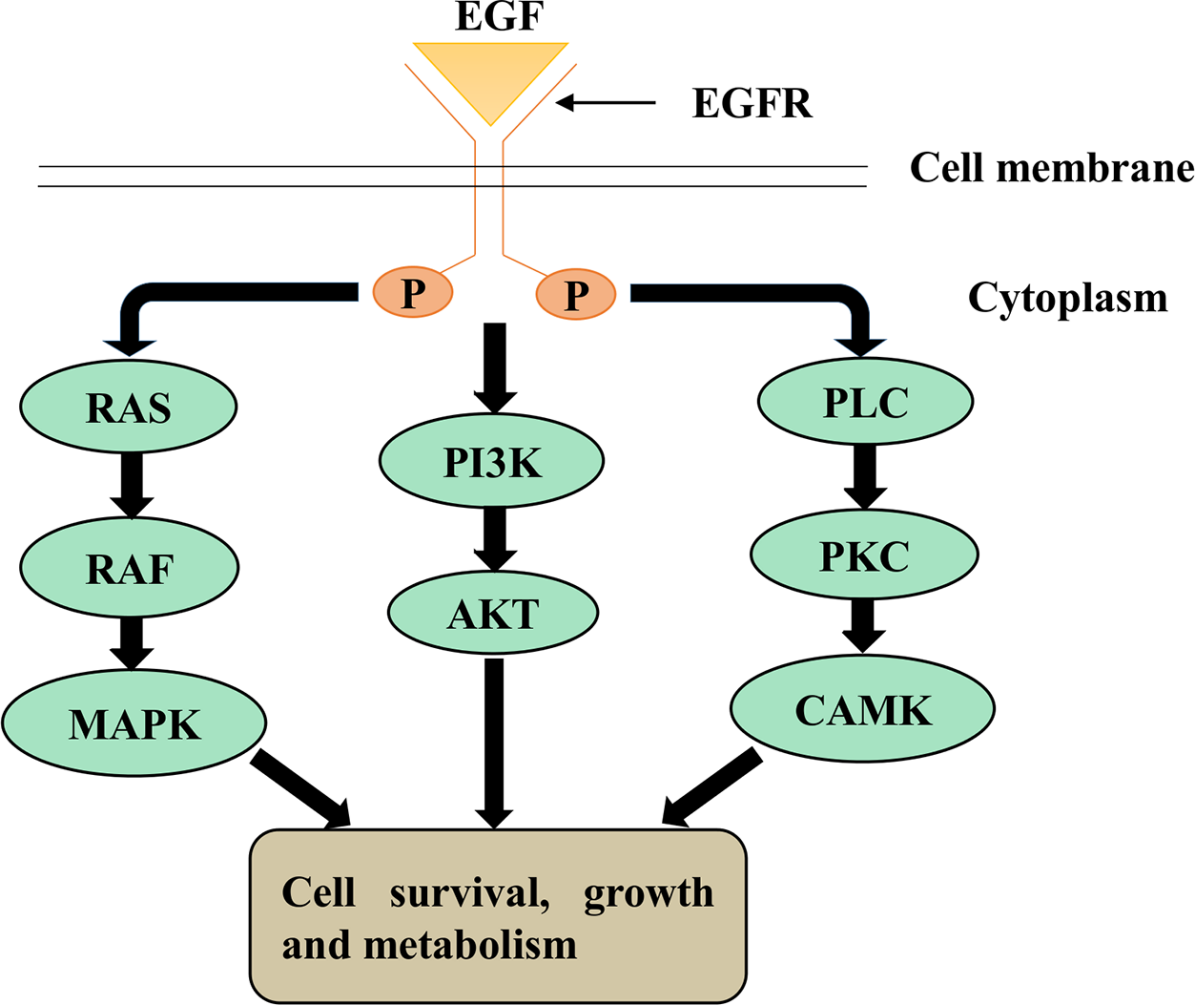
An overview of downstream signaling pathways induced by EGFR signaling. Three main pathways: AKT, RAS/MAPK and CaM and DAG/IP3 are induced downstream of active EGFR signaling.

EMT is induced by a number of distinct molecular processes [1]. These include the binding of several growth factors, including the platelet derived growth factor (PDGF), insulin-like growth factor (IGF), neuregulin and epidermal growth factor (EGF) to their cognate cell-surface receptors, leading to receptor activation [4]. This activates downstream signaling pathways that regulate the control of specific transcription factors, cell-surface proteins and microRNAs [5]. EMT is also involved in reorganization and expression of cytoskeletal proteins and production of ECM-degrading enzymes [1]. This series of events leads to increased expression of mesenchymal markers like N-cadherin and vimentin and decreased expression of epithelial markers such as E-cadherin [6]. Binding of EGF to its cognate epidermal growth factor receptor (EGFR) family has been shown to stimulate EMT in breast cancer cells [7,8], leading to altered expression of E-cadherin and vimentin [8,9]. Activated EGFR signaling suppresses E-cadherin expression either by promoting its endocytosis [10] or by enhancing the expression of transcription factors (TFs) like Snail and Twist [11,12]. As a result, the cells may transition from epithelial to mesenchymal phenotype with spindle like morphology [8]. EGFR regulates mammary gland development and in certain aggressive breast cancer cells has been shown to regulate invasion and migration [8]. The most common signaling cascades activated downstream of EGFR are PI3K/Akt, Ras/Raf/Mek and DAG/IP3 and CaM signaling, that affect cell cycle progression, inhibition of apoptosis, angiogenesis, tumor cell motility, and metastases **(Fig 1)** [13,14].

EMT is likely to impact metabolism, but the effects are not as widely studied as cancer metabolism [15,16] Cancer cells exhibit a shift of ATP generation from oxidative phosphorylation to aerobic glycolysis known as the Warburg effect [17]. This leads to a higher rate of glycolysis in cancer cells. Cancer cells also tend to show enhanced glutamine metabolism which has been shown to contribute cancer cell migration [18]. Signaling pathways have often been associated with metabolic consequences, but can themselves be influenced by metabolism. Interestingly, up-regulated glycolysis has been linked with higher AKT signaling in cancer cells [19,20]. However, the mechanistic manner in which metabolism is affected during EMT is unknown.

Computational approaches such as Constraint-based modeling and analysis (COBRA) techniques are very useful in analysis of the complex biological networks like signaling networks [21–23]. Prior efforts of modeling of signaling cascades include modeling of the EGFR pathway [21,22,24–26], TLR signaling [27–29], JAK-STAT signaling [23], MAPK pathway [30] and interleukin 1 signaling [31]. COBRA techniques mainly focus on the use of physio-chemical and biological constraints and are sparsely dependent on kinetic data that has limited availability. Protocols for the generation of biochemical networks and computational algorithms/methods for querying these networks are now well established [32,33]. They involve conversion of biological data (e.g. genomic, metabolic, and regulatory) to a mathematical reaction format. This allows better definition of regulatory changes associated with specific events such as EMT and exploration of metabolic alterations associated with the process. For example, how altered EGFR signaling is propagated to a metabolic phenotype can be investigated using COBRA methodology.

In this study, we built a computational signaling network of EGFR to query how the expression of signaling genes can affect metabolic alterations during EMT in human breast epithelial cells. An EGFR signalling network was reconstructed (EGFR_SN) and was constrained with the transcriptomics data of the breast epithelial cell line D492 and its mesenchymal counterpart D492M [34] to form EGFR_E and EGFR_M networks. D492 is an E6/E7 viral oncogene immortalized human breast epithelial basal cell line with stem cell like properties that differentiates into both myoepithelial and luminal cells and has the ability to undergo branching morphogenesis when grown in a 3D reconstituted basement membrane matrix [35]. The 3D co-culture of D492 cells with human endothelial cells led to establishment of mesenchymal cells with spindle-like morphology called D492M. The D492M cells have high expression of N-cadherin and vimentin and low expression of E-cadherin that is typical for cells that have undergone EMT [34].

The EMT specific signaling network of D492 and D492M enabled us to investigate differential gene expression of EGFR signalling genes. This was subsequently extended to other breast epithelial cell lines and their mesenchymal counterparts. Through extensive literature review, the EGFR_SN network was linked to metabolic genes that were likely to be affected. The differential flux values in EGFR_E and EGFR_M allowed the assessment of how the altered signaling affects metabolic gene expression and metabolism in D492 and D492M cells. Increased flux within the AKT and RAS/MAPK signaling pathways was predicted in D492 as compared to D492M. The *in silico* predicted increase in flux of the AKT pathway induced higher glycolytic activity in D492 cells. This suggested that there may be an EMT-related decrease in glycolysis in D492M as compared to D492 cells, which was confirmed *in vitro* by glucose uptake and lactate secretion measurements. Comparative analysis of EGFR signaling networks in three other breast epithelial cell lines showed that regulation of signaling pathways are cell specific and follow no simple trend.

## Results and Discussion

### EGFR signaling network reconstruction

In order to capture how altered EGFR signaling is propagated through metabolic pathways in the breast epithelium, we built a constraint based EGFR network, EGFR_SN. To generate EGFR_SN, the EGFR pathway map (Reactome ID: R-HSA-177929) was downloaded from the Reactome database [36]. Several modifications such as incorporation of gene-protein reaction (GPRs) rules/association, additions of modifiers, inhibitors and activators and removal of dead ends were made to the Reactome pathway to make it feasible for analysis with the COBRA methodology (methods section). Due to the incorporated GPRs, experimental data (e.g, gene expression, proteomic, fluxomic data) can now be mapped onto EGFR_SN, thereby relating genomic information to the reactions in the network.

**Fig 2:**
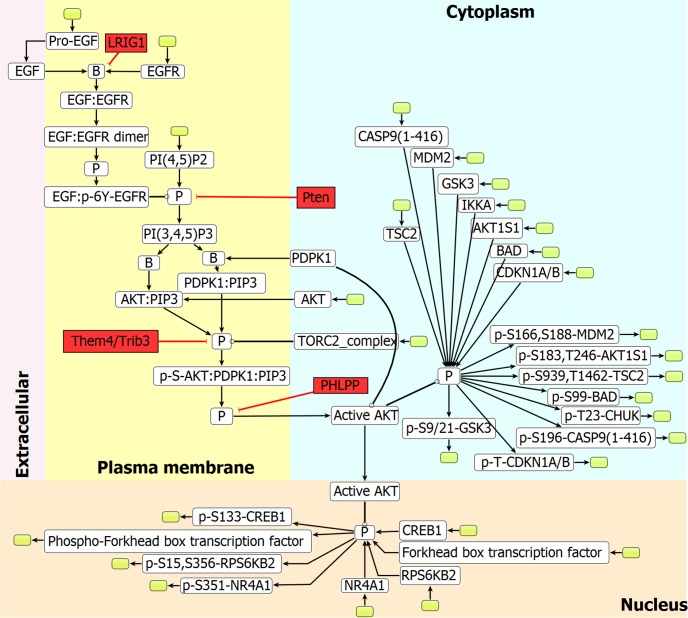
Sub-network of EGFR_SN network. A part of the EGFR signaling network representing AKT signaling. Nodes indicate the reacting component and edges denote the reactions. Red nodes indicate inhibitors which were manually added to the model in the form of GPRs (information obtained from the Reactome database), white nodes indicate signaling components present in the original SBML file downloaded from the Reactome database and green nodes indicate exchange reactions which were added to the model to remove dead ends. Node ‘B’ and ‘P’ indicates binding, and phosphorylation, respectively. Edges: → transition, ⟞ inhibition, ⫯ catalysis.

The resulting reconstructed network EGFR_SN accounts for 182 reactions, 216 genes, 152 reacting species, 11 inhibitors and 2 activators. The 182 reactions were divided between 83 internal reactions and 99 exchange reactions. Exchange reactions were added in order to remove dead-ends in the network and acquire feasibility. The number of exchange reactions added here is large compared to what is typically present in metabolic networks, where exchange reactions are added mainly to allow for accumulation of metabolites and secretion of wastes. Internal reactions represent connections between internal signaling components, while the exchange reactions represent connections of the system boundary with the environment. **Fig 2** shows a sub-network of EGFR_SN representing AKT signaling.

### D492 and D492M specific EGFR signaling networks

Microarray gene expression profiles of the human breast epithelial cell line D492 and the mesenchymal like D492M were used to constrain the EGFR_SN network to build an EMT specific signaling model. D492 can be used as a model for studying EMT for which biological data is available: mRNA, micro-RNA, cell phenotypic data, growth curves etc. [34,37]. D492 has previously been used in determining the role of microRNAs in EMT [37], studies related to branching morphogenesis [38] and more recently it was used to investigate the role of EGFR as a tumor suppressor [39].

**Fig 3:**
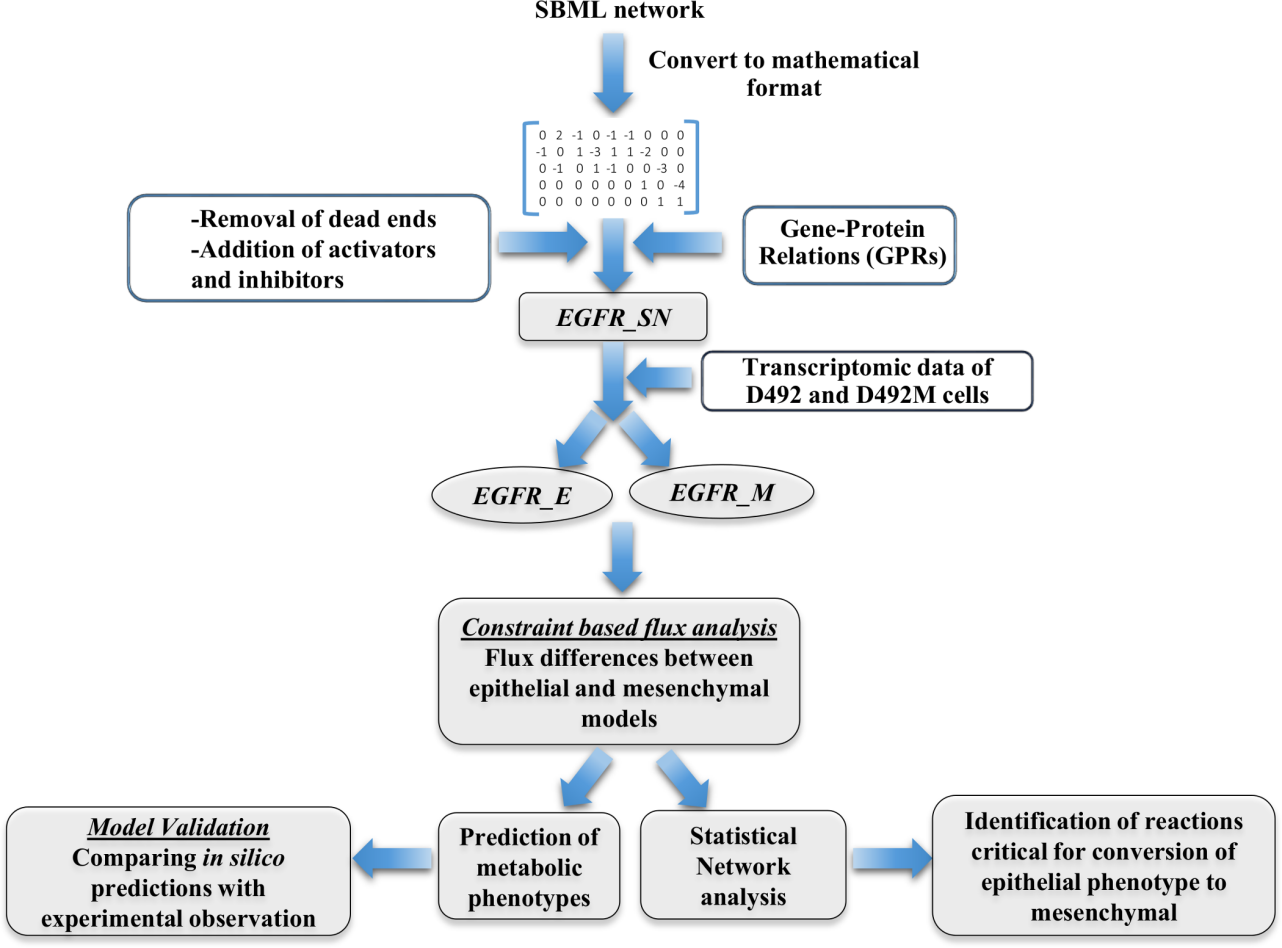
The pipeline employed to generate context specific signaling networks EGFR_E and EGFR_M of an immortalized breast epithelial cell line (D492) and mesenchymal cell line (D492M). SBML pathway map from Reactome was converted to mathematical format using the COBRA toolbox. This network was further modified to remove dead ends and include GPRs for modifiers, activators and inhibitors, resulting in the formation of the EGFR_SN network. The EGFR_SN network was constrained with the transcriptomic data of D492 and D492M to form EGFR_E and EGFR_M networks. Flux differences between EGFR_E and EGFR_M were used to further predict metabolic phenotype and target reactions critical for the reversal of mesenchymal to epithelial phenotype.

To derive epithelial and mesenchymal specific signaling networks, gene expression data from D492 and D492M was mapped onto the EGFR_SN network. The pipeline is described in **Fig 3**. Differentially regulated (up-regulated and down-regulated) genes in the two cell lines led to two different signaling networks: EGFR_E and EGFR_M for D492 and D492M respectively, as described in the methods section.

### Network simulation of D492 and D492M

#### Flux analysis discriminates between D492 and D492M EGFR signaling phenotypes

**Fig 4:**
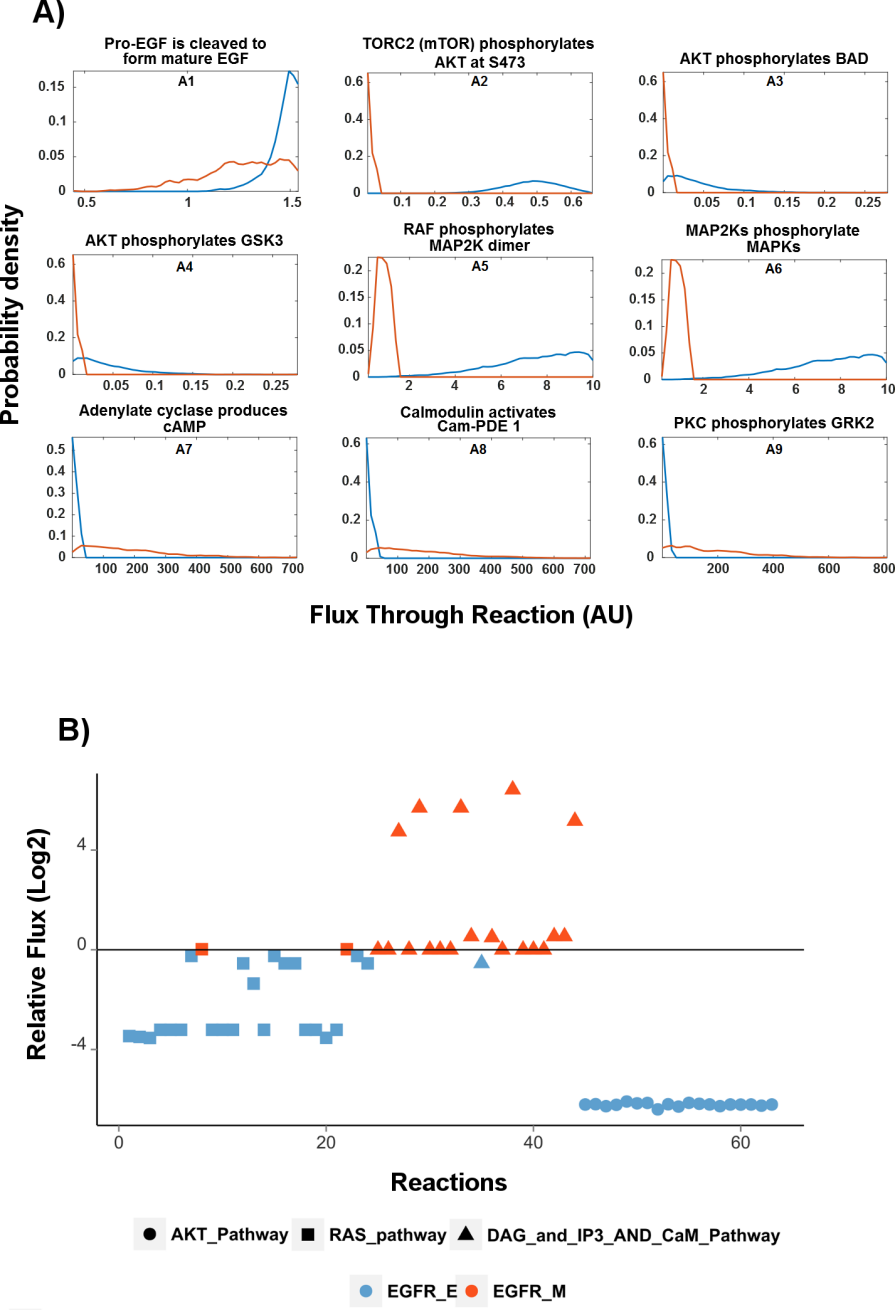
Flux differences between epithelial network (EGFR_E) and mesenchymal network (EGFR_M). A) Probability density estimates for the flux values in selected reactions as obtained by random sampling. The blue curve represents the flux distribution of EGFR_E and the red curve that of EGFR_M. Vertical axis denote probability and flux values are represented on the horizontal axis. AU: arbitrary units B) Relative mean flux for each reaction in EGFR_E and EGFR_M through the AKT, RAS and DAG/IP3 and CaM pathways. Higher flux within reactions in AKT and RAS/MAPK pathways are observed in the EGFR_E network compared to EGFR_M while CaM and DAG/IP3 have higher flux in EGFR_M. Negative values denote higher flux in EGFR_E and positive values denotes higher flux in EGFR_M. Numerical values of these fluxes are given in supplementary file (S1 File).

EGFR_E and EGFR_M networks were investigated through flux analysis. Flux distribution in EGFR_E and EGFR_M networks were estimated using random sampling [21,40] (Fig 4). Fig 4A shows the probabilty density for the flux in selected reactions, where red and blue lines denote EGFR_M and EGFR_E, respectively. Fig 4A, panel A1 shows that the most probable flux for this reaction in EGFR_E is higher than in EGFR_M, indicating this reaction is more active in EGFR_E. The panels A2-A6 belonging to AKT and RAS/MAPK pathways show that the most probable flux in EGFR_M is lower than the most probable flux in EGFR_E. The graphs in panels A7-A9, belong to the CaM pathway and are in contrast to panels A2-A4, indicating higher flux in EGFR_M.

Fig 4B shows the relative mean flux values in each reaction within the AKT, RAS/MAPK and DAG/IP3 and CaM pathways. The EGFR_E network had higher flux through the AKT and RAS/MAPK pathways. In contrast, the transition from epithelial (EGFR_E) to mesenchymal (EGFR_M) phenotype promotes higher flux through the calcium CaM signaling pathway in the EGFR_M network. Moreover, flux was also increased in the di-acyl glycerol (DAG) and inositol trisphosphate (IP3) pathway in the EGFR_M network, which constitutes an important part of the CaM pathway.

### Predicting reversal of mesenchymal to epithelial phenotype

We next studied how the flux in the EGFR_M network could be modified so that it became similar to the EGFR_E flux, in order to identify how the EMT process could be reversed. This was achieved by using an optimization algorithm similar to the MOMA algorithm which is frequently used to study perturbations in metabolic networks [41]. The algorithm searches for a flux distribution in EGFR_M, which most closely resembles the average flux values obtained previously for the EGFR_E model with random sampling, with minimum relaxation in the reaction bounds in EGFR_M while maintaining steady state conditions, see the methods section for more details.

The algorithm highlighted five reactions whose bounds needed to be relaxed such that the flux distribution of EGFR_M is close to that of EGFR_E. These included three internal reactions: 1) phosphorylation of the MAP2K dimer by RAF, 2) phosphorylation of MAPKs by MAP2Ks (RAF/MAPK pathway), 3) PIP2 conversion to PIP3 by PIK3 (AKT pathway), and two exchange reactions, belonging to the RAF/MAPK pathway. Through GPRs we identified that there are 22 genes associated with the reactions predicted above **(S1 Table)**. However, among these 22 genes, *MAPK1*, *NRAS*, *HRAS and EGFR* genes were overexpressed in D492 as compared to D492M and the inhibitor *PTEN* was overexpressed in D492M.

Based on these predictions and by analysing the flux differences between EGFR_E and EGFR_M, we hypothesize that increased AKT and RAS/MAPK signaling in D492 epithelial cells promotes their transition to the D492M mesenchymal like phenotype. However, after attainment of a mesenchymal state, AKT and RAS/MAPK signaling is reduced and alternative pathways such as CaM signaling gets activated which may be involved in the maintenance of the mesenchymal state. We also hypothesize that up-regulation of *MAPK1*, *NRAS*, *HRAS and EGFR* and down-regulation of *PTEN* inhibitor in EGFR_M may lead to its transformation into EGFR_E.

**Fig 5:**
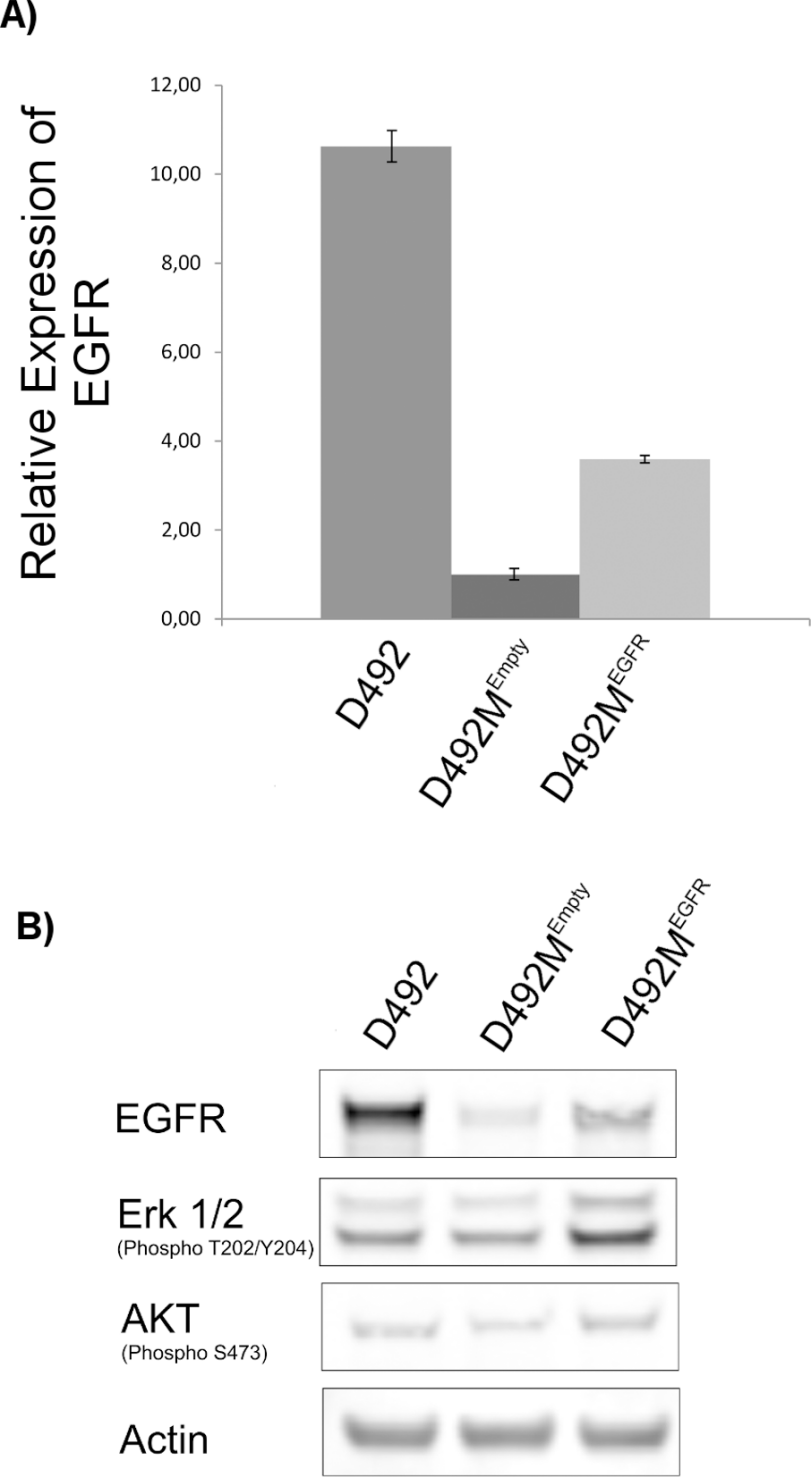
EGFR overexpression in D492M. **(A)** Real-Time Quantitative Reverse Transcription PCR of EGFR in D492, D492M^EGFR^ and D492M^Empty^, normalized to GAPDH. EGFR transcription level is significantly higher in D492M^EGFR^ compared to D492M^Empty^ but does not reach the D492 EGFR transcription level. **(B)** Protein expression of EGFR, Phospho-p44/42 MAPK (ERK1/2) and Phospho-AKT determined by Western blotting. Overexpression of EGFR in D492M leads to increased protein expression of EGFR in D492M^EGFR^ compared to D492M^Empty^, but EGFR protein expression does not reach the D492 level. Overexpression of EGFR in D492M leads to increased MAPK (Erk1/2) and Akt phosphorylation which is higher than in both D492M^Empty^ and D492.

Interestingly, most of the *in silico* predicted targets from our study have previously been implicated to play a role in EMT. The activation of MAPK1 protein has been shown to induce EMT in MCF10 breast epithelial cells by phosphorylation and consequent stabilization of Twist1 [42]. In another study, the silencing of MAPK1 led to increased expression of E-cadherin and a decrease in vimentin and Snail expression in human cervical cancer cells [43], suggesting its role in the transition from epithelial to mesenchymal phenotype. HRAS and Slug together have been shown to induce the expression of vimentin and enhance cell migration in pre-malignant MCF10A breast epithelial cells [43]. Further, activation of EGFR has been shown to induce the expression of Twist by activating STAT3, suggesting a prominent role of EGFR in EMT [11]. In a recent study where mesenchymal cells were generated from D492 by overexpressing HER2 (ErBb2) [39], subsequent overexpression of EGFR promoted mesenchymal to epithelial transition. In light of this finding, and based on our *in silico* predictions, we made EGFR overexpressing D492M cell line (D492M^EGFR^) **(method section)**. D492M^EGFR^ cells showed higher phosphorylation of AKT and ERK1/2 **(Fig 5)**, although reversal of mesenchymal to epithelial phenotype was not observed **(S1 Fig)**. Thus, we hypothesize that for complete reversal of mesenchymal to epithelial phenotype, the *MAPK1*, *NRAS and HRAS* genes may need to be overexpressed in addition to EGFR, along with *PTEN* inhibition in D492M. Likewise, maintaining expression of the *MAPK1*, *NRAS*, *HRAS and EGFR* in epithelial cells while suppressing the expression of *PTEN* is expected to inhibit EMT. At present, a number of EGFR inhibitors have been approved by the FDA for cancer treatment. These include Cetuximab and Panitumumab that are monoclonal antibodies against EGFR, and Erlotinib, Gefitinib and Lapatinib which are specific tyrosine kinase inhibitors against EGFR. All these compounds are prescribed against advanced, late-stage or metastatic cancers [44,45]. The findings we present here suggest that inhibition of EGFR may contribute to or increase the risk of EMT in cancers of epithelial origin. Indeed, the overexpression of HER2 in D492 cells has recently been shown to suppress EGFR expression and induce EMT [39]. Our predictions also indicate that *PTEN* inhibition can help maintain the epithelial phenotype, thereby preventing EMT and metastasis. However, recent findings suggest that loss of PTEN function may promote tumor progression in a mouse model [46] and EMT in human colon cancer cells [47], highlighting the fact that further research on the role of *PTEN* in EMT is needed.

Taken together, these above results demonstrate the EGFR_SN network can be used to study in detail the differences in EGFR signaling between epithelial and mesenchymal cells during EMT and identify gene targets which could possibly be used to hinder or even revert EMT.

### Crosstalk of the signaling and metabolism in D492 and D492M

We next studied how changes in AKT signaling could influence metabolic gene expression in EMT. The AKT pathway was chosen since it has previously been shown to affect various metabolic pathways, e.g. glycolysis and fatty acid metabolism in cancer cells [19,48]. To reflect how alterations in the AKT signaling pathway may affect metabolism, a literature based survey was conducted to identify the connections between this pathway and its target metabolic genes. This suggested that active AKT signaling induces the expression of metabolic genes involved in glycolysis, fatty acid metabolism and purine and pyrimidine metabolism, while it suppresses the expression of metabolic genes involved in gluconeogenesis (**Table 1)**.

**Table 1:** Predicted expression of metabolic genes regulated by AKT in D492 and D492M cells.

No.	References	Metabolic Genes	Proposed Expression	Microarray Expression
1	[50]	*GAPDH (Glyceraldehyde-3-phosphate dehydrogenase)*	↓ M	↓ M
2	[51]	*GLUT1 (facilitated glucose transporter)*	↓ M	↓ E
3	[48,52]	*GYS1 (Glycogen [starch] synthase*, *muscle)*	↓ M	↓ M
4	[53,54]	*HK1 (Hexokinase-1)*	↓ M	↓ M
5	[53,54]	*HK2 (Hexokinase-2)*	↓ M	↓ M
6	[52]	*G6PC (Glucose-6-phosphatase)*	↓ E	NA
7	[52]	*PCK1 (Phosphoenolpyruvate carboxykinase 1)*	↓ E	NA
8	[48]	*ACLY (ATP-citrate synthase)*	↓ M	↓ M
9	[48]	*ME1 (Malic enzyme)*	↓ M	↓ M
10	[19,55]	*PFKFB2 (6-phosphofructo-2-kinase/fructose-2*,*6-bisphosphatase 2)*	↓ M	NA
11	[48]	*HMGCR (3-hydroxy-3-methylglutaryl coenzyme A reductase)*	↓ M	↓ M
12	[48]	*HMGCS1 (Hydroxymethylglutaryl-CoA synthase*, *cytoplasmic)*	↓ M	↓ M
13	[56]	*ACC (acetyl-CoA carboxylase alpha)*	↓ M	NA
14	[56]	*SREBF1 (Sterol regulatory element-binding protein 1)*	↓ M	↓ E
15	[48,56]	*SREBF2 (Sterol regulatory element-binding protein 2)*	↓ M	NA
16	[56]	*FASN (Fatty acid synthase)*	↓ M	↓ M
17	[57]	*ATIC (Bifunctional purine biosynthesis protein PURH)*	↓ M	↓ M
18	[57]	*HPRT1 (Hypoxanthine-guanine phosphoribosyltransferase)*	↓ M	↓ M
19	[57]	*TALDO1 (Transaldolase)*	↓ M	↓ M
20	[58]	*TKT (Transketolase)*	↓ M	↓ M

Increased flux through the AKT pathway in EGFR_E network as compared to EGFR_M suggested up-regulated expression of genes involved in glycolysis, fatty acid and purine/pyrimidine metabolism in D492 cells and down-regulated expression of genes involved in gluconeogenesis pathway. The “References” column lists the studies from which the influence of AKT signaling on the expression of the corresponding metabolic genes was derived. No: 1–7 belong to Carbohydrate metabolism, 8–16 to fatty acid metabolism and 17–20 to purine/pyrimidine metabolism. NA: gene expression data is not present in the microarray data set.

The ratio of average flux in each signaling reaction of the AKT pathways in the EGFR_M and EGFR_E network was used to identify differences in relative metabolic gene expression in the D492 and D492M cells. The higher flux observed in the AKT pathway in the EGFR_E network suggested increased expression of genes belonging to glycolysis, fatty acid and purine/pyrimidine metabolism in D492 in comparison to D492M (**Table 1**). Higher expression of glycolytic genes suggested higher glycolytic activity in the D492 cells.

To test these *in silico* predictions, we compared the predicted expression of the metabolic genes with their corresponding relative expression values in the microarray data set [34] and compared relative expression of metabolic genes in D492 and D492M. Up- and down-regulated metabolic genes were identified based on differential expression and significance measurements, as analyzed by SAM [49], included in the microarray dataset. A cut-off of 0.05 on the significance measure was used. The relative gene expression of 13 out of 15 metabolic genes (86.6%) affected by AKT signaling were in agreement with the predicted *in silico* expression (**Table 1)**. Since the experimental expression values of the metabolic genes were not used to generate the EGFR_E and EGFR_M networks, this data corresponds to an independent validation set for the *in silico* predictions.

However, the gene expression of 2 out of 15 (13.3%) of the metabolic genes (*GLUT1* and *SREBF1)* from the microarray data and *in silico* predictions were not in agreement. This suggests an alternate level of regulation that metabolic genes may encounter during EMT, in addition to the direct regulation by molecular signaling pathways. For accuracy measure we did not take into account metabolic genes which did not have any detectable expression values (denoted *NA*). Ultimately, we observed that the expression of most of the metabolic genes directly affected by AKT signaling during EMT was correctly predicted in our D492 model system.

### Glycolytic flux is increased in D492 as compared to D492M and consistent with model predictions

**Fig 6:**
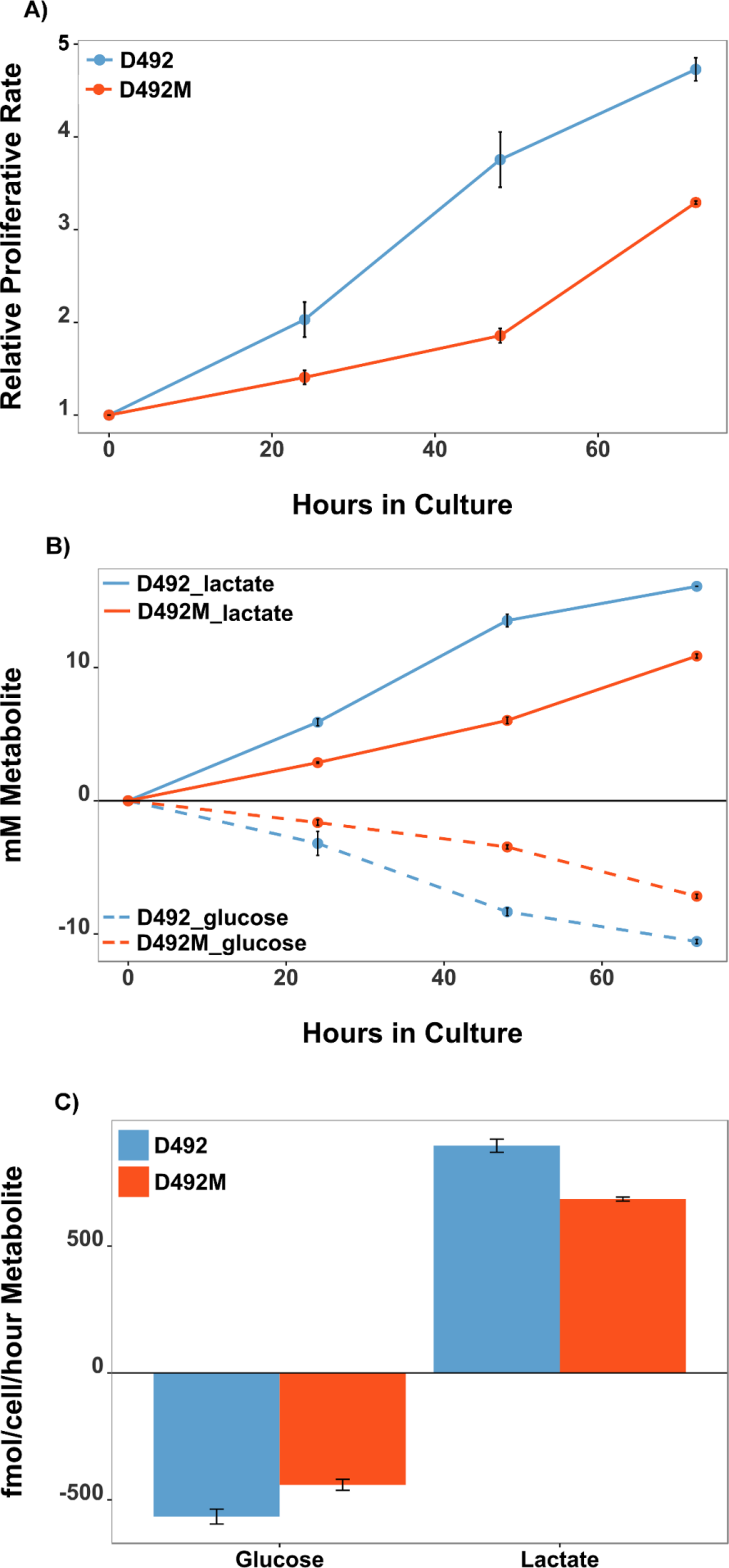
Proliferation rate and glycolytic activity are higher in D492 than in D492M cells. A) Cell proliferation assay demonstrated a higher growth rate of D492 cells compared to D492M cells. B) Spent medium analysis of glucose (dashed lines) and lactate (solid lines) shows higher glucose uptake and lactate secretion in D492 cells (blue) than in D492M cells (red). C) Calculated glucose uptake and lactate secretion rates indicate higher glycolytic flux rates per cell per hour in D492 cells than D492M cells. Data represents results from 3 independent experiments. Error bars represent standard deviation in a single experiment done in triplicate. mM: milli molar, fmol: femto molar.

Gene expression measurements of metabolic genes are not necessarily a quantitative predictor of their metabolic activity. We therefore tested our *in silico* prediction that glycolytic activity is higher in D492 than in D492M by measuring the proliferation rates and glucose consumption and lactate secretion rate to estimate glycolytic activity *in vitro*. Cells with a higher rate of proliferation may have higher nutrient and energy requirements and consequently greater metabolic demand [59]. Cell proliferation assays showed that the D492 cells had a higher growth rate than D492M **(Fig 6A).** Spent medium of D492 and D492M cells was analyzed to measure glucose and lactate levels. Higher glucose levels and lower lactate levels in cultured supernatants of the D492M cells indicated a lower rate of glucose consumption and lactate secretion rates in D492M cells **(Fig 6B)**. This indicates a lower glycolytic rate in D492M cells compared to D492 and suggests a shift in the glycolytic capacity of the cells in response to EMT and is in agreement with the *in silico* predictions that indicated higher gene expression of glycolytic enzymes in D492 cells.

We conclude that this EMT related decrease in aerobic glycolysis appears to be driven by an overall decrease in the expression of glycolytic enzymes due to down-regulated AKT signaling in D492M cells. To the best of our knowledge, this is the first report that demonstrates that *in silico* network predictions can be used to study the influence of a molecular signaling pathways, such as AKT, on the metabolic outcome during EMT in breast epithelial cells. Our findings are in agreement with a previous study on human non-small cell lung carcinoma (NSCLC) cells [60] which demonstrated a decrease in aerobic glycolysis during EMT but are in contrast to results obtained for MCF7 breast epithelial cells that have undergone EMT [61].

### EMT metabolic network constrained with AKT signaling regulated metabolic genes

Results from the previous section showed that the metabolic phenotype could be accurately predicted by *in silico* analyses of the changes in the expression of AKT signaling genes. Further, we investigated how these changes in AKT signaling, that impact the expression of metabolic genes (**Table 1**), are propagated through other metabolic pathways during EMT. Based on RECON2 [62], we have also built an EMT metabolic network (MODEL1602080000). This metabolic network was built by constraining RECON2 with microarray gene expression data [34] of metabolic genes during EMT in D492 and D492M. RECON2 is a global human metabolic reconstruction that has been used previously to investigate regulation of metabolism in diseases like obesity and diabetes [40,62]. In this study, we constrained our EMT metabolic network with the metabolic genes that were predicted to be regulated by changes in AKT signaling (**Table 1**). This led to the formation of epithelial metabolic (Met_E) and mesenchymal metabolic (Met_M) networks, specific for AKT signaling regulated metabolism during EMT (described in methods section). Metabolic differences between the Met_E and Met_M models were identified based on differences in their relative flux span (methods section). Reactions carrying higher flux in Met_E compared to Met_M included reactions that are involved in N-glycan metabolism, Glycolysis, Fatty acid synthesis, Fatty acid oxidation, nucleotide interconversion and pentose phosphate pathway. Reactions carrying higher flux in Met_M involved Glutathione, glycerophospholipid, and inositol phosphate metabolism. Alteration of metabolic pathways, including N-glycan, glutathione metabolism, glycolysis, fatty acid and purine metabolism that we observed from our constructed Met_E and Met_M metabolic networks, have been shown to play an important role in the regulation of EMT [19,48,57,60,63,64]. However, the details of the mechanism are still unknown. A list of the metabolic reactions similarly predicted to be affected by the AKT pathway in the Met_E and Met_M networks are provided in the supplementary file **(S2 File)**. In conclusion, this method was able to predict metabolic pathways that may be affected downstream upon activation of AKT signaling in breast epithelial cells during EMT. This method extends the approach of associating metabolic phenotype with regulation of signaling pathways. Further, it also suggests the possibility of determining the metabolic regulation in cases that are limited by the availability of metabolomic data or the gene expression data of metabolic genes. Although these predictions are context specific in relation to AKT signaling, the integration of other signaling pathways affected during EMT may give a more coherent picture of altered metabolism.

### Comparative analysis of the EGFR signaling network between D492 and three other human breast epithelial cell lines

**Fig 7:**
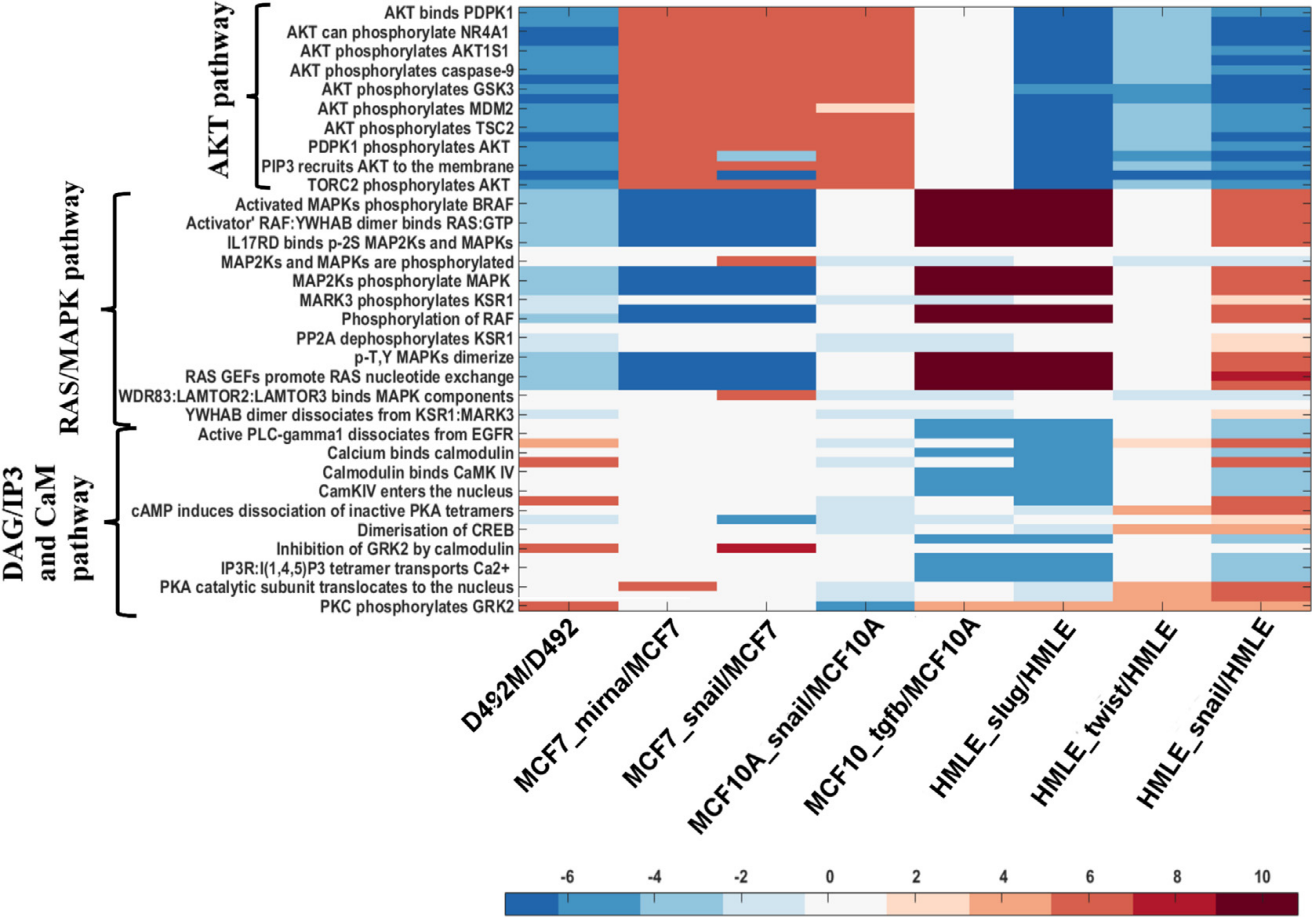
Comparison of flux distribution in the reactions within AKT, RAS and CaM pathway in different breast epithelial cell lines. The heat map has been generated using log2 relative mean flux of the reactions within the AKT, RAS and DAG/IP3 and CaM pathways in epithelial and mesenchymal cells of D492, MCF, MCF10A and HMLE cells. Exchange reactions are not included in the heat map. The negative and positive values as depicted on the color scale denote higher flux in reactions of epithelial and mesenchymal cells, respectively. D492M, MCF7_mirna, MCF7_snail, MCF10_snail, MCF10A_tgfb, HMLE_slug, HMLE_twist and HMLE_snail are EMT derived mesenchymal cells.

We next studied whether a general trend of higher flux in the reactions of the AKT and RAS pathways (downstream of EGFR) was observed in other human breast epithelial cell lines, when compared to their mesenchymal counterparts. We mapped the microarray transcriptomic datasets for the three human breast epithelial cell lines (HMLE, MCF-7 and MCF-10A) onto the EGFR_SN network similar to the method used for the D492 cells (methods section). Comparisons between cell lines were done in terms of the ratio between flux in the mesenchymal network vs flux in the corresponding epithelial network **(Fig 7)**. Numerical values of the fluxes within AKT, RAS and CaM pathways in HMLE, MCF-7 and MCF-10A are given in supplementary file **(S3 File)**.

Higher flux through the reactions in the AKT pathway was observed in both the D492 and HMLE epithelial cells, suggesting that the HMLE cells may have similar metabolic phenotype as D492 **(Fig 7)**. In contrast, the mesenchymal counterparts of MCF7 and MCF10A cells had higher flux in the AKT pathway **(Fig 7),** suggesting that cells that have undergone EMT may have increased glycolytic activity. This is in agreement with Kondaveeti *et al*. who have reported an increase in glycolytic activity post-EMT in MCF-7 cells as a result of increased expression of glucose transporters and lactate dehydrogenase [61]. The flux in the RAS/MAPK pathway was higher in the D492 and the MCF7 epithelial cells than in their mesenchymal counterparts. Analysis of DAG/IP3 and CaM pathway showed that D492M, MCF7 mesenchymal cells and those of HMLE which have undergone EMT due to induction of Twist, had higher flux as compared to their epithelial counterpart **(Fig 7)**. Thus, no general pattern was observed in the flux distributions between the epithelial and mesenchymal networks for the different breast epithelial cell lines. Induction of EMT by different factors (viral induction of SNAIL, SLUG, TWIST, miR374a or TGFβ1 treatment) also seemed to differentially regulate signaling pathways as was evident in the MCF10A and HMLE cells. For example, the induction of EMT in HMLE cells by overexpression of Slug and Twist resulted in different flux patterns in the RAS/MAPK and DAG/IP3 and CaM pathways **(Fig 7)**. Similar effect was seen by Deshiere *et al*., where they have shown that TGFβ1 treatment and CK2b silencing activate divergent signaling pathways, that ultimately lead to EMT in MCF-10A cells [65]. Finally, we compared the predicted metabolic phenotypes to the metabolic gene expression data for the MCF7, MCF10A and HMLE cell lines similar to the method used for the D492 cells, however the gene expression data of metabolic genes were not statistically significant and hence was not included in our study **(S1–S4 Tables)**.

In summary, we observed that different cell lines may affect different signaling regulation during EMT. Moreover, variation in the methods of EMT induction may dictate differential regulation of the signaling and metabolic cross talk.

### Conclusions

Herein we have demonstrated a method to build a stoichiometric model of the EGFR signaling (EGFR_SN) network employing COBRA methods that aids in understanding the differential activation of downstream EGFR signaling pathways during EMT. Epithelial and mesenchymal specific EGFR signaling networks were obtained by integrating microarray transcriptomics data of signaling genes from the D492 breast epithelial and mesenchymal cells with EGFR_SN. The epithelial and mesenchymal networks were used to predict the expression of metabolic genes. The predicted expression values were in agreement with transcriptomics data of metabolic genes as well as biochemical data that demonstrated higher glycolytic activity in D492 epithelial cells. Furthermore, signaling genes leading to reversion to the epithelial phenotype (MET) via up- regulation in the mesenchymal cells were predicted. Additional *in vitro* testing would be required to confirm these *in silico* predictions. Thus, in this study we showed that the metabolic phenotype can be predicted *in silico* using gene expression profiles of EGFR signaling components.

EGFR_SN is not limited in scope to the investigation of EMT. The signalling network could be used to highlight signaling-metabolic crosstalk in different cell types for which metabolic reconstructions exist [66] or for different conditions [66] where EGFR signalling is known to be influential [67]. Furthermore, the network described herein could be expanded to allow for more comprehensive coverage of signalling pathways of relevance to EMT. Signaling networks for platelet derived growth factor (PDGF), insulin like growth factor (IGF), and vascular endothelial growth factor (VEGF) for example, could be constructed and co-integrated. The co-integration of these signaling networks regulated by different growth factors would give more comprehensive knowledge of cross talk between signaling and metabolic pathways during EMT.

The pipeline developed for D492 was used on other cell models representative of breast epithelial cell lines. We assumed that different breast epithelial cells models would have similar signaling patterns and hence could have a general interpretation of regulation of signaling pathways during EMT. In contrast to our hypothesis, the regulation of signaling pathways showed no general pattern and appeared to be a cell-specific phenomenon. Flux values in the AKT pathway from our network, suggest that there may be an EMT related decrease in aerobic glycolysis in both the HMLE and D492 cell lines, while the opposite was observed in the MCF-7 and MCF-10A breast epithelial cells. There are several possible explanations for this disparity. First, the highly complex nature of the regulation of EMT, which may be differentially regulated by the cellular micro environment or EMT inducing factors as seen in our *in silico* predictions. Second, our study of EMT signal transduction is primarily based on transcriptional signatures which may or may not necessarily correlate with the translational output (protein-levels) [68,69]. This last issue might be addressed by co-integrating transcriptomics and proteomics/phospho-proteomics data in order to obtain more accurate models. Such a strategy was recently reported where it was used to reconstruct a metabolic network for predicting of metabolic signatures in diabetes patients [70]. The disparity between cell models suggests considerable heterogeneity of the cell models used for EMT research in general. Finally, these efforts highlight a lack of comprehensive datasets available that accurately describe EMT and ultimately hinder mechanistic understanding of the genotype phenotype relationship underlying EMT. The direct link between regulation of signaling pathways and the consequent metabolic phenotype may be of clinical interest, as metabolically based therapeutics to combat cancer EMT could be masked by inaccurate metabolic understanding.

## Methods

### Reconstruction of signaling network

The EGFR pathway network was downloaded from the Reactome database which is curated and peer reviewed [71]. The EGFR pathway was then converted from SBML to COBRA format for further analyses. This conversion is based on the stoichiometric coefficients of the reacting species provided in the Reactome pathway and also requires setting constraints on each reaction in the form of lower and upper bounds, which determine the minimum and maximum allowable reaction rates (fluxes), respectively [33]. Flux in a signaling network is defined as the rates of phosphorylation, de-phosphorylation, dimerization, or binding of proteins.

The COBRA model was represented by an *m* by *r* stoichiometric matrix ***S***, where *m* denotes the number of network components (metabolites, proteins, and complexes) and *r* the number of network reactions. Reactions within the network were mass-balanced. The system was assumed to be at steady state, which means that the fluxes **v** = (v_1_,…,v_m_) satisfy the equations **Sv**  =  **0**. The upper and lower bounds of all the internal reactions were set to 1000 and zero, respectively. A lower bound of zero was used since all the reactions are irreversible.

The initial COBRA model contained many dead ends and was infeasible due to network gaps [72]. Dead-ends represents those reacting species which are either only produced or only consumed in the network, leading to blocked reactions, i.e. reactions unable to carry flux.To remove the dead ends and obtain a feasible model, exchange reactions were introduced allowing uptake and secretion of components across the system boundary. By adding exchange reaction for all reacting species, a feasible model was obtained. In this model, the network topology becomes irrelevant since all demands on the network can be met by the exchange reactions. To avoid this situation, an optimization algorithm, ‘relax_rxns’ **(S1 Dataset)** was developed that enabled a feasible steady state network, while minimizing uptake/secretion (exchange) of the dead-end molecules. The methodology is similar to the one used by Vardi *et al*. [21]. First, the lower bounds of all the internal reactions were set to 1 to force removal of blocked reactions (reactions with zero flux) and consequently removal of the dead-end species. Exchange reactions were then added for every reacting species in the network, initially with all uptake and secretion rates set to zero. The optimization algorithm returns a minimal set of exchange reactions that need to be present in order to remove all dead ends. These reactions were included in the final model, but the other exchange reactions were removed. The optimization problem was formulated as follows:

$$\text{minimize}\;\sum_{\text{j}\in {\text{R}}_{\text{r}}}\;{\text{y}}_{\text{j}}$$ (1)

Sv=0 (2)

$${\text{l}}_{\text{j}}\;\text{-}\;{\text{n}}_{\text{j}}\leq {\text{v}}_{\text{j}}\leq {\text{u}}_{\text{j}}+{\text{p}}_{\text{j}}\;\text{j}\in {\text{R}}_{\text{r}}$$ (3)

$${\text{l}}_{\text{i}}\leq {\text{v}}_{\text{i}}\leq {\text{u}}_{\text{i}}\;\text{i}\in {\text{R}}_{\text{n}}$$ (4)

$${\text{p}}_{\text{j}}\leq {\text{My}}_{\text{j}},\;{\text{n}}_{\text{j}}\leq {\text{My}}_{\text{j}}\;\text{j}\in {\text{R}}_{\text{r}}$$ (5)

$${\text{p}}_{\text{j}}\geq 0,\;{\text{n}}_{\text{j}}\geq 0\;\text{j}\in {\text{R}}_{\text{r}}$$ (6)

$${\text{y}}_{\text{j}}\in \left\{0,1\right\}\;\text{j}\in {\text{R}}_{\text{r}}$$ (7)

The decision variables are **v**, the flux values in individual reactions, p_j_ and n_j_ which represent the amount of relaxation of upper and lower bounds for reaction *j*, respectively and binary variables y_j_ which indicate whether reaction *j* is relaxed or not. The objective is to minimize the number of reactions that are relaxed (1). The steady state mass balance constraints are represented by (2), R_r_ is the set of reactions that are to be relaxed (3) with the corresponding upper and lower bounds set to zero. The set R_n_ represents all the remaining reactions (4) with the corresponding upper bounds set to 1000 and lower bounds set to 1. The value of the constant M in (5) was set to 1000. The optimization model was implemented in Matlab (Mathworks, Natick, MA, USA) using the CVX modeling language [73,74] and solved using the Gurobi solver [75]. The version of CVX used in this study supports binary variables as those in constraint (7).

### Modelling modifiers, activators and inhibitors

The signaling network from section 4.1 was extended to include modifiers, activators and inhibitors. Modifiers are phosphorylated protein entities that further phosphorylate downstream targets. In the original Reactome pathway, the modifiers were not included as reacting species in reactions, such that they were not connected to downstream targets. For this reason modifiers were included as the reacting species in their target reactions. We included the modifiers in the same way as described by Dasika *et al*. [22]. Briefly, modifier mod1 acts as a modifier for the transition of A to B. In order to avoid unambiguous stoichiometry, we added mod1p, as a product of mod1 during the reaction as shown below.

Production of mod1→mod1(exchange reaction)

A+mod1→B+mod1p(mod1becomes mod1p)

mod1p→consumption of mod1p(exchange reaction)

Activators and inhibitors are responsible for positive and negative regulation of the reaction, respectively. Activators and inhibitors were included in the model via gene-protein reaction rules as described in the next section.

### Addition of gene information

**Fig 8:**
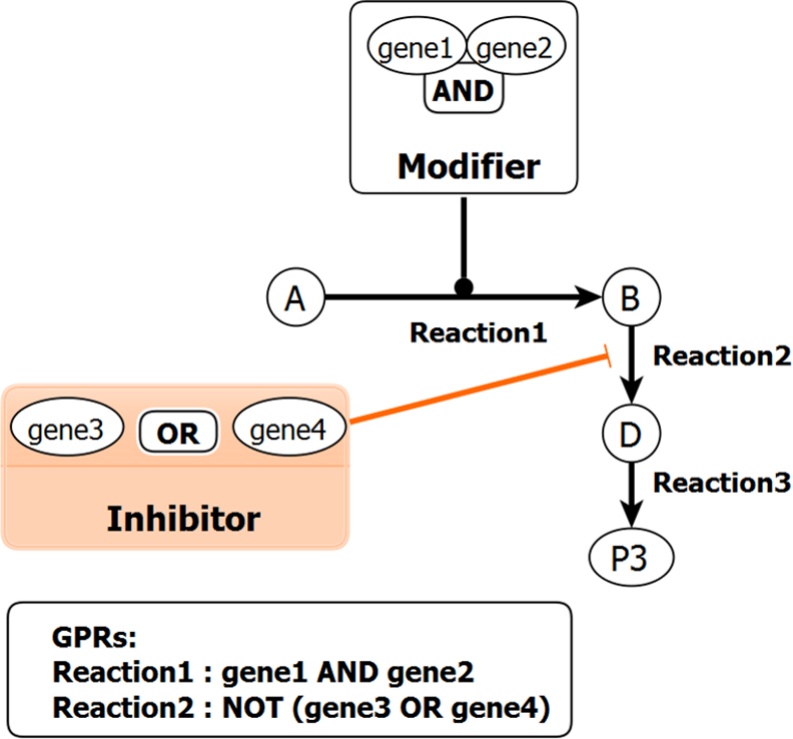
The rules for adding GPRs. Reaction1 is catalyzed by a modifier which is a multiprotein complex of gene1 and gene2. The combination of both the genes are required for the reaction to take place. Reaction2 is inhibited in presence of inhibitor which is an isoform of gene3 and gene4. Presence of either gene3 or gene4 will inhibit Reaction2. A ‘NOT’ operator is assigned to the GPR of Reaction2 to indicate inhibition. A GPR is not assigned to Reaction 3 in this example.

The original Reactome signaling network described the transmission of signal, in terms of activation or inhibition of the subsequent downstream entities. This did not include any enzymatic reactions or the gene-protein rules required to map gene expression data [33]. The signaling network was therefore modified to include GPRs by associating each network reaction with genes encoding modifiers, activators and inhibitors. Reactions not having any information of modifiers, activators or inhibitors were not designated with GPRs. For the GPR generation, we employed UniProt IDs of the protein entities within the Reactome pathway to identify genes, which were then associated with reactions using Boolean logic. Multiprotein complexes were represented with an ‘AND’ operator, while isoforms were represented by an ‘OR’ operator to create a corresponding Boolean rule. Wherever an inhibitor was involved, the corresponding genes were prefixed by a ‘NOT’ operator in the GPR. Exchange reactions were also assigned GPRs. If a reaction is catalysed by a modifier and inhibited by an inhibitor, the presence of modifier will activate the reaction, while an inhibitor will inhibit the reaction. GPRs for all the reactions are provided in the S1 File in the sheet named “GPRs”. The blank rows denote that they were not assigned any GPRs. **Fig 8** below, illustrates examples of GPR generation.

The resulting network is referred to as EGFR_SN. A spreadsheet containing all the network reactions, reacting species, modifiers, inhibitor, activators, and GPRs in different sheets is provided in the supplementary file **(S1 File).** All the models used in this study are provided in the supplementary dataset **(S1 Dataset)**.

### Microarray data integration

Gene expression data for the D492 and D492M cell lines was obtained from Sigurdsson *et al* [34]. The microarray expression data for the HMLE, MCF-7 and MCF-10A cell lines was obtained from NCBI GEO [76], GEO IDs: GSE52593 [77], GSE43495 [78], GSE58252 [79], GSE39358 [80], and GSE28569 [65]. Illumina/Affimetrix IDs within the microarray data were mapped with the Uniprot IDs of the genes in the EGFR_SN network using the Python API of bioDBnet, biological DataBase network. Mapped gene expression of the EGFR signaling genes for each cell line is provided in the supplementary material S4 File with sheets named after each cell line. Negative values indicate higher gene expression in epithelial cells and positive values indicate higher gene expression in mesenchymal cells. Integration of expression data with the EGFR_SN network and subsequent analysis was performed in Matlab. The cut-off value on the relative fold change between epithelial and mesenchymal cell lines, to determine up- and down-regulated genes, depended on the relative expression of housekeeping genes and the statistical significance (p-value ≤ 0.05) of the fold change. Accordingly, a cut-off of ≥ 2 was considered for D492 cells, 0.5 for MCF7 and MCF10A cells and 0.3 for HMLE cells. Since GPRs link genes with reactions, up- and down-regulated genes identified consequently up-regulated and down-regulated reactions. The change in the expression value of each gene in the EGFR_SN signaling network was used to define the upper and lower flux bounds of its associated reactions, such that up-regulated reactions were allowed to have higher flux values and down-regulated reactions were allowed to have lower flux values. Since, the upper bounds on individual fluxes are essentially infinite, up-regulation in an epithelial model was simulated by downregulating the corresponding reaction in its mesenchymal counterpart, and vice versa. Flux bounds of the up-regulated reactions in D492 were constrained by an arbitrary factor one-hundredth of the initial bounds in D492M and vice versa. Similarly, flux bounds of the activated and inhibited reactions were constrained. Activation in the epithelial model was simulated by downregulating the corresponding reaction in its mesenchymal counterpart and vice versa, while inhibition was simulated by downregulating the corresponding reaction in the same model. This led to the formation of EGFR_E and EGFR_M networks for D492 and D492M respectively.

Random sampling was used to obtain flux distributions in the networks [81] using the COBRA toolbox [32], technical details of which can be found in Supplementary methods **(S1 Methods)**.

### Flux differences in epithelial and mesenchymal networks and crosstalk to metabolism

Flux differences in individual reactions in the mesenchymal and epithelial networks were quantified in terms of fold changes, v_M_(i) / v_E_(i) where v_M_(i) and v_E_(i) represent the average flux in reaction i for the mesenchymal and epithelial networks, respectively. Reactions which had v_M_(i) / v_E_(i) greater than 1 carried higher flux in the mesenchymal network. A ratio below 1 indicates higher flux in the epithelial network. A literature based survey provided evidence of whether a metabolic gene is positively or negatively regulated by AKT signaling **(S5 Table)**. The prediction of the metabolic gene expression derives from whether EGFR_E or EGFR_M have higher flux in AKT signaling.

### Minimization of the distance between the Mesenchymal and Epithelial flux distributions

The optimization algorithm of section 4.1 was modified by replacing the objective function (1) by

$$\text{minimize}\;\alpha \|\text{v}–{\text{v}}_{\text{E}}\|+(1\text{-}\alpha )\sum_{\text{j}\in {\text{R}}_{\text{r}}}\;{\text{y}}_{\text{j}}$$ (1a)

where v and y_j_ represents the decision variables as before, v_E_ are fixed values representing the mean flux distribution of EGFR_E, obtained from random sampling and ||. || represents the Euclidean norm. The upper and lower bounds for each flux correspond to the values from the EGFR_M network. The algorithm returns a set of reactions in EGFR_M whose bounds can be relaxed in order to obtain a flux distribution that resembles that of EGFR_E.

### The effects of active AKT signaling on the EMT metabolic network

**Fig 9:**
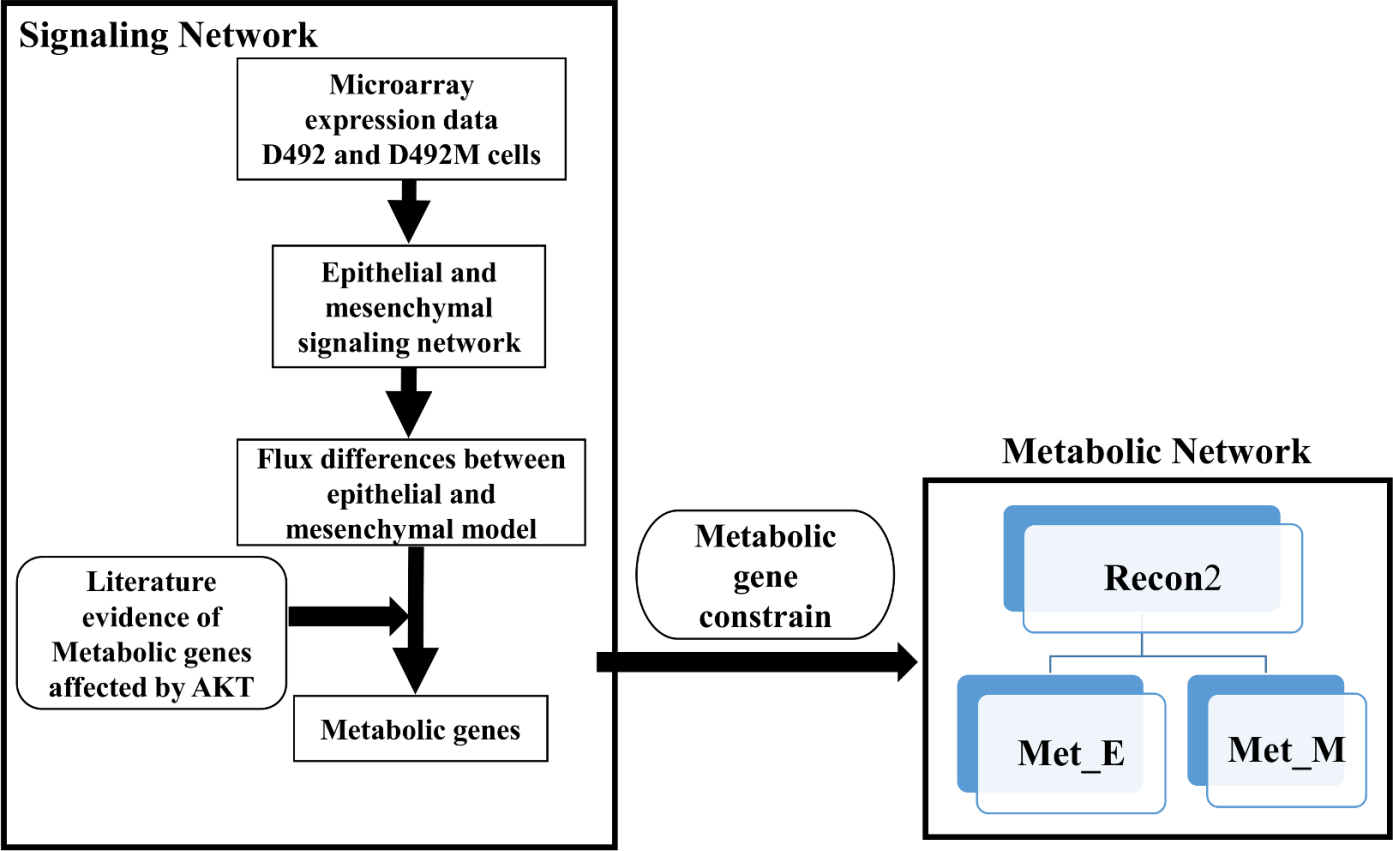
The pipeline used to determine the effects of active AKT signaling on the EMT metabolic network. The metabolic genes predicted to be altered in D492 and D492M dependent on the AKT signaling network, were used to constrain the EMT metabolic network generated from Recon2, in order to identify downstream metabolic pathways that are affected by AKT activation.

We constrained RECON 2 [62] using the microarray data from Sigurdsson *et al*. [34] to generate an EMT metabolic network (submitted to Biomodels: MODEL1602080000). This reconstruction consists of all the metabolic reactions encoded in both the D492 and D492M cell lines. This EMT metabolic network has information on GPRs connecting each reaction with the genes of the enzymes catalyzing the reaction. Since GPRs associate genes with corresponding reactions, the metabolic genes predicted to be up-regulated in epithelial cells due to AKT signaling (**Table 1**) led to the identification of up-regulated reactions in D492 and similarly were determined in D492M. This information was then used to define the upper and lower flux bounds of the affected reactions in the EMT metabolic network to form Met_E (metabolic epithelial) and Met_M (metabolic mesenchymal) networks **(Fig 9)**. Up- and down-regulation of the Met_E and Met_M models was simulated as described in section 4.4. Specifically, up-regulation in an epithelial metabolic model was simulated by downregulating the corresponding reaction in its mesenchymal counterpart, and vice versa. Flux bounds of the up-regulated metabolic reactions due to AKT signaling in D492 were constrained by an arbitrary factor, one-hundredth of the initial bounds in D492M and vice versa.

The flux values through each reaction in both the models were determined through random sampling method and the relative flux span, s_M_(i)/ s_E_(i) was used to quantify the flux differences between the networks. Here, s_M_(i) and s_E_(i) represent the average flux in reaction i for the mesenchymal and epithelial cells, respectively.

### Cell proliferation assay and metabolite measurements

For proliferation assays and spent medium analysis, 1.5 × 10^4^ D492 or D492M cells were cultured in 48-well plates (Costar) in 200μL H14 medium as previously described [35]. Spent medium was collected and cells were fixed in ice-cold methanol at 0 h, 24 h, 48 h and 72 h. To estimate proliferation, cells were stained with crystal violet, washed thoroughly with water, dissolved in 30% acetic acid and read in a spectrophotometer at 570 nm. The observed growth rates were 0.0276 h^−1^ for D492 and 0.0161 h^−1^ for D492M. The glucose and lactate concentrations in the spent medium were measured in an ABL90 blood gas analyser (Radiometer, Brønshøj, Denmark). Glucose uptake and lactate secretion per cell were calculated for each cell line as described in [82], based on the ABL90 measurements and growth rates.

### Viral transduction

Vectors used for viral production were acquired from Addgene, pBABE-EGFR and empty backbone (#11011, #1764, respectively) and were used as provided. Phoenix HEK293 cells were used for retroviral (EGFR) virus production, using Arrestin transfection (Life Technologies). D492M cells were transduced overnight with viral supernatant containing 8 μg/ml Polybrene (Sigma-Aldrich). EGFR and empty backbone cells were selected using 2 μg/ml puromycin (Life Technologies).

### Real-Time Quantitative Reverse Transcription PCR

Total RNA was isolated using TRI-Reagent solution (Ambion) and reverse transcribed using SuperScript IV (Invitrogen). The resulting cDNA was used for Real-Time Quantitative Reverse Transcription PCR, in Maxima Probe/ROX qPCR Master Mix (Thermo Scientific) with primer pairs and probes for *EGFR* (Hs00540086_m1, Life Technologies), *ZEB1* (Hs00232783_m1, Life Technologies) and *GAPDH* (Hs99999905_m1, Life Technologies). Experiments were done in triplicates on 7500 Real Time PCR System (Applied Biosystems). EGFR mRNA levels were normalized to *GAPDH* and relative mRNA differences were calculated using the 2^ΔCt^ method.

### Western blotting

Proteins were isolated using RIPA lysis buffer supplemented with protease and phosphatase inhibitors (Life Technologies). For Western blot analysis 5 μg of protein lysates were loaded per lane on NuPage 10% Bis-Tris gels (Life Technologies) in 2-(N-morpholino)ethanesulfonic acid (MES) running buffer (Life Technologies). Samples were denatured using 10% mercaptoethanol at 95°C for 10 minutes before loading. Samples were transferred to Immobilon FL PVDF membranes (Millipore) and blocked in Li-cor blocking buffer for 1 hour. Primary antibodies were incubated overnight at 4°C and secondary IRDye antibodies were incubated at room temperature for 1 hour (Licor). The following primary antibodies were used for Western blotting: Actin antibody (Abcam, ab3280), EGF Receptor (Cell Signaling, CS#4267), Phospho-Akt (Ser473) (Cell Signaling, CS#4060), Phospho-p44/42 MAPK (Erk1/2) (Thr202/Tyr204) (Cell Signaling, CS#4370), N-Cadherin (BD Biosciences, 610921), E-Cadherin (BD Biosciences, 610182) and Cytokeratin 14 (Abcam, ab15461). Near-infrared fluorescence visualization was measured using Odyssey CLx scanner (Li-Cor, Cambridge, UK).

## Supporting Information

S1 Dataset Codes and all the models in COBRA format used in this study. (ZIP)

S1 File A spreadsheet describing EGFR_SN attributes, along with flux distribution in EGFR_E and EGFR_M for D492 cells. (XLS)

S2 File Flux distribution in Met_E and Met_M. (XLS)

S3 File Flux distribution within EGFR signaling cascade in MCF7, MCF10A and HMLE cell lines. (XLSX)

S4 File Expression data mapped to EGFR signaling genes for each cell line. (XLSX)

S1 Methods Supplementary methods. (DOCX)

S2 Methods Modelpipeline: Detailed procedure for generation and simulation of EGFR signaling network. (DOCX)

S1 Fig Epithelial and mesenchymal marker expression. **(A)** Western blotting for epithelial markers E-Cadherin and CK14 and mesenchymal marker N-Cadherin. Overexpression of EGFR in D492M does not revert the mesenchymal phenotype towards an epithelial phenotype. D492M^EGFR^ retains N-Cadherin expression and does not gain E-Cadherin or CK14 expression. **(B)** Real-Time Quantitative Reverse Transcription PCR of the EMT transcription factor ZEB1 in D492, D492M^EGFR^ and D492M^Empty^ normalized to GAPDH. ZEB1 transcription was not detected in D492 and the transcription level of ZEB1 was unchanged in D492M^EGFR^ compared to D492M^Empty^. D492M^EGFR^ retains mesenchymal ZEB1 expression.
(TIFF)

S1 Table Gene associated with the reactions important for the reversal of EGFR_M to EGFR_E. (DOCX)

S2 Table Predicted expression of metabolic genes regulated by AKT in HMLE cells. ER: Microarray Expression data in TWIST, SLUG and SNAIL induced HMLE cells respectively. PE: Proposed Expression. Predictions in agreement with microarray data are highlighted in green and that otherwise are highlighted in orange.
(DOCX)

S3 Table Predicted expression of metabolic genes regulated by AKT in MCF10A cells. ER: Microarray Expression data in TWIST, SLUG and SNAIL induced HMLE cells respectively. PE: Proposed Expression. Predictions in agreement with microarray data are highlighted in green and that otherwise are highlighted in orange.
(DOCX)

S4 Table Predicted expression of metabolic genes regulated by AKT in MCF7 cells. ER: Microarray Expression data in TWIST, SLUG and SNAIL induced HMLE cells respectively. PE: Proposed Expression. Predictions in agreement with microarray data are highlighted in green and that otherwise are highlighted in orange.
(DOCX)

S5 Table Regulation of metabolic gene expression by AKT signaling. “Reference” column lists the studies from which the influence of AKT signaling on the expression of the corresponding metabolic genes was derived. +1 and -1 denotes positive and negative regulation, respectively.
(DOCX)

S1 Appendix Full form of abbreviations. (DOCX)
