# Supplementary material for: EGFR Signal-Network Reconstruction Demonstrates Metabolic Crosstalk in EMT
Source: PLoS Comput Biol. 2016 Jun 2;12(6):e1004924. doi: 10.1371/journal.pcbi.1004924 (PMC4890760; doi:10.1371/journal.pcbi.1004924)
Supplement: S1 Methods — (DOCX) [file pcbi.1004924.s006.docx]

# Supplementary material and methods

In the following, COBRA toolbox functions are denoted by italic font, Matlab code and variables by a monospaced font.

## Random Sampling

Flux distribution in signaling and metabolic network was calculated by Random Sampling method. The random sampling method obtains points that are uniformly distributed in the region of allowed solutions and calculates the most probabilistic steady-state flux value for each reaction [1]. Random sampling was performed by the *gpSampler* function [2] in the COBRA toolbox which returns two outputs, ‘sampleStructOut’ and ‘mixedFraction’.

[sampleStructOut, mixedFraction] = gpSampler(model, npts, [], dur);

Where, sampleStructOut is a structure containing sampling results in the matrix ‘sample points’ and mixedFraction determines the quality of the sampling. A mixed fraction of 0.5 indicates uniform sampling of the solution space. ‘npts’ is the sample points, ‘dur’ is the duration of sampling.

For signaling networks, 1000 sample points were used (as they were smaller network), in contrast, for metabolic network, 5000 sampling points were used. Sampling was run for 2 hrs for signaling networks, while metabolic network was allowed to run for 24 hrs.

For visualization of random sampling results as shown in **Figure 4,** *plotSampleHist* function was used.

plotSampleHist (rxnList, {sampleE.points, sampleM.points},{EGFR_E, EGFR_M}, nbins, perScreen)

Where, *rxnList* is the list of reactions to be plotted, sampleE and sampleM are the sampling structure of EGFR_E and EGFR_M models respectively, nbins is the number of bins to be used and perScreen is the number of reactions to be shown on the screen.

## Optimization algorithm

The optimization algorithm ‘relax_rxns’ returns five outputs. The algorithm includes a constant “alpha”, as a fraction between 0 and 1. One can choose to set the value of alpha to be 0 for addition of minimum set of exchanges and any value between 0 < alpha < 1 to minimize the distance between EGFR_E and EGFR_M. ‘relaxed_model’ is the feasible model in case of alpha = 0 or the model with closer flux distribution to the ‘target_flux’ in case of alpha =1).

### For addition of exchanges:

The lower bound of all the internal reactions were set to zero and then exchanges for every reacting species in the network with their bounds set to zero as shown below

model.lb (findRxnIDs(model,model.rxns)) = 1;

modelEx = model;

metList = model.mets;

for i=1:length(metList)

modelEx = addExchangeRxn(modelEx, metList(i), 0, 0);

end

[relaxed_model, ~, ~, ~, rxns_relaxed] = relax_rxns(modelEx, strmatch('Ex_', modelEx.rxns),[], length(modelEx.rxns), [], 0);

rxns_relaxed are the exchange reactions which are needed for the relaxed_model to carry flux. Additional exchanges added initially which are not relaxed by the algorithm were removed.

### For minimization of distance (reversal of mesenchymal to epithelial):

[relaxed_model, ~, d, ~, rxns_relaxed] = relax_rxns_new(EGFR_M,[],[],[],VE, alpha);

Where d is the distance of the flux distribution in relaxed_model and the target_flux. Target flux here is VE, which is flux distribution in EGFR_E (epithelial network), alpha is the value between 0 < alpha < 1

For ex if we want to revert mesenchymal EGFR_M to EGFR_E:

[relaxed_model, ~, d, ~, rxns_relaxed] = relax_rxns(EGFR_M,[],[],[],VE,0.9);

“relaxed_model“ is the reversed model which is identical to EGFR_E. VE is the target flux, i.e the flux of the epithelial model EGFR_E. rxns_relaxed highlights the five reactions whose flux bounds needs to be altered in order to change EGFR_M to EGFR_E.

**References**

1. Schellenberger J, Palsson BØ. Use of randomized sampling for analysis of metabolic networks. J Biol Chem. 2009;284: 5457–61. doi:10.1074/jbc.R800048200

2. Schellenberger J, Que R, Fleming RMT, Thiele I, Orth JD, Feist AM, et al. Quantitative prediction of cellular metabolism with constraint-based models: the COBRA Toolbox v2.0. Nat Protoc. 2011;6: 1290–307. doi:10.1038/nprot.2011.308
