# Supplementary material for: EGFR Signal-Network Reconstruction Demonstrates Metabolic Crosstalk in EMT
Source: PLoS Comput Biol. 2016 Jun 2;12(6):e1004924. doi: 10.1371/journal.pcbi.1004924 (PMC4890760; doi:10.1371/journal.pcbi.1004924)
Supplement: S2 Methods — (DOCX) [file pcbi.1004924.s007.docx]

Supplementary material and methods

**Procedure/Pipeline for model construction and simulation**

In the following, COBRA toolbox functions are denoted by italic font, Matlab code and variables by a monospaced font.

1. **Download the Reactome pathway in SBML format**.
2. **Convert pathway to COBRA format** using *convertSBMLtoCOBRA*. The resulting model, stored in variable model was infeasible and unable to carry flux because of dead ends.
3. **Exchange reactions were added** to remove dead end from the reactions. First, the lower bounds of all the internal reactions were set to one and then exchanges for every reacting species in the network were added. The upper and lower bounds for the exchanges were initially set to zero:

model.lb (findRxnIDs(model,model.rxns)) = 1;

modelEx = model;

for i=1:length(model.mets)

modelEx = addExchangeRxn(modelEx, model.mets(i), 0, 0);

end

where model is the infeasible model and modelEx is the model with exchanges added for all the reacting species.

The function ‘relax_rxns’ provided in the supplementary material ‘S1_Dataset’ under the folder named ‘code’, was then run on modelEx:

[relaxed_model, ~, ~, ~, rxns_relaxed] = relax_rxns(modelEx, strmatch('Ex_', modelEx.rxns),[], length(modelEx.rxns), [], 0);

rxns_relaxed is the minimal set of exchange reactions needed for the model to carry flux and relaxed_model is the final model in which bounds of the minimal set have been relaxed. Exchanges which were not relaxed by the algorithm were then removed by the *removeRxn* function.

1. **Addition of modifiers to the COBRA model.** We added modifiers by including reactions as follows:

Production of mod1 → mod1 (exchange reaction)

A + mod1 → B + mod1p (mod1 becomes mod1p)

mod1p → consumption of mod1p (exchange reaction)

A list of modifiers is provided in the supplementary file ‘S1_File’ under the sheet ‘modifiers’.

1. **Model testing:** Flux variability analysis was used to test whether the model was now able to carry flux.
2. **Model Trimming:** The original pathway included 350 proteins and 3 DNA/RNAs. Some of the reactions were loop reactions which do not result in useful flux predictions under steady state conditions. This suggests that all loop reactions should be removed. Also, most reactions in the original SBML file that were removed in EGFR_SN network were series of reactions which led to the production of inhibitors. One of the assumption in steady state modeling is mass conservation, meaning that the total amount of any reacting species being produced is equal to the amount being consumed. This ultimately eliminates the possibility of estimating the effect of inhibitors on the reactions of the network. Hence, we removed these inhibitor reactions and added inhibitors manually in the model in Gene Protein Reaction (GPR) rules. The inhibition was taken into account by constraining the upper and lower bounds of the reactions. In this manner, the model was able to include the effect of inhibitors on the network reactions. The Reactome database provides a PDF file describing positive and negative regulation of reactions. This file was carefully reviewed while taking inhibitors into account. Activators were similarly added manually in the model in GPRs.
3. **Addition of gene information (GPRs):** GPRs describe the relationship between genes, proteins and reactions in the form of Boolean logic. Multiprotein complexes are represented with an ‘AND’ operator, while isoforms are represented by an ‘OR’ operator. Similar strategy was used to determine the GPRs of the reactions in the signaling network by associating reactions with genes encoding modifiers, activators and inhibitors. Wherever an inhibitor was involved, the corresponding genes were prefixed by a ‘NOT’ operator in the GPR.

Reactome has information about Uniprot Ids of each entity. We manually checked for each entity whether they were multi-protein complexes or an isoform and generated a corresponding Boolean rule. For e.g. a reaction that is catalyzed by a modifier which is a multiprotein complex of gene1 and gene2, the latter of which is a isoform, would have GPR as : gene1 AND (gene2a or gene2b), gene2a and gene2b are the isoforms of gene2. More examples are illustrated in **Figure 8**.

We have provided the GPRs for each reaction in S1_File in the sheet named “GPRs”. Blank rows indicate that no GPRs were assigned. The modified model was named EGFR_SN and is provided in the supplementary file ‘S1_Dataset’.

1. **Microarray data integration:** The transcriptomic data for D492 and D492M was downloaded from Sigurdsson *et al* [1]. The microarray expression data for the HMLE, MCF-7 and MCF-10A cell lines was obtained from NCBI GEO [2], GEO IDs: GSE52593 [3], GSE43495 [4], GSE58252 [5], GSE39358 [6], and GSE28569 [7]. The standard transcriptomic data has Ilumina/Affimetrix IDs or NCBI Gene Ids. The genes in our model were represented by Uniprot IDs. In order to map the gene expression values, we converted the Uniprot Ids to Ilumina/Affimetrix IDs or NCBI Gene Ids in the transcriptomics data using the Python API of bioDBnet, biological DataBase network (https://biodbnet-abcc.ncifcrf.gov/webServices/SoapSampleCode.php). In this way we were able to identify the gene expression values of the signaling genes in our model. We have updated the supplementary material S4_File with different sheets containing the mapped gene expression data for each cell line. Negative and positive values indicate higher gene expression in epithelial cells and mesenchymal cells, respectively. For some cell lines, such as D492, the data set did not have detectable expression values for few of the genes, in this case, the expression was assumed to be absent and was ignored. Duplicates values were present for some of the genes, e.g. in sheet “MCF7_mirna”. In those cases, the highest expression values were selected.

We subsequently used these expression values to determine the upregulated and downregulated genes in the epithelial and mesenchymal cells.

1. The Matlab script ‘createE&M’ used to identify up- and down-regulated genes, affected reactions and to create EGFR_E and EGFR_M is provided in the supplementary material ‘S1_Dataset’ in the folder ‘code’.
2. **Flux calculation:** Random sampling was used to obtain flux distributions in the networks [8] using the COBRA toolbox [9] and the following command:

[sampleStructOut, mixedFraction] = gpSampler(model, npts, [], dur);

Where, sampleStructOut is a structure containing sampling results in the matrix ‘sample points’ and mixedFraction determines the quality of the sampling. A mixed fraction of 0.5 indicates uniform sampling of the solution space. npts (1000) is the number of sample points, dur (2 hrs) is the duration of sampling. Additional technical details are provided in the supplementary material S1_Methods.

The average flux distribution is calculated by taking mean of the ‘sample points’ in sampleStructOut.

VE = mean (sampleE_points, 2), where VE is the mean flux distribution and sampleE_points is the matrix containing possible flux solutions.

Results of random sampling and mean flux values for each model are provided in the supplementary material S1_Dataset under the folder named ‘results’. ‘results’ folder has multiple .mat files for each cell lines observed in this manuscript. Flux distribution is also included in the supplementary spreadsheet S1_File and S3_File.

1. **Flux differences in the epithelial and mesenchymal networks and metabolic crosstalk:** Flux differences in individual reactions in the mesenchymal and epithelial networks were quantified in terms of fold changes, v_M_(i) / v_E_(i) where v_M_(i) and v_E_(i) represent the average flux in reaction i for the mesenchymal and epithelial networks, respectively. Reactions which had v_M_(i) / v_E_(i) greater than 1 carried higher flux in the mesenchymal network. A ratio below 1 indicates higher flux in the epithelial network. A literature based survey provided evidence of whether a metabolic gene is positively or negatively regulated by AKT signaling **(S5_Table)**. The prediction of the metabolic gene expression derives from whether EGFR_E or EGFR_M have higher flux in AKT signaling. The length of the VE and VM vectors corresponds to the number of reactions in the model and can be queried by the reaction index number, E.g: 'RAF phosphorylates MAP2K dimer' is first reaction in the model, and its flux in epithelial network can be queried by ‘VE(1)’.
2. **Model simulation to predict reversal of mesenchymal to epithelial phenotype:**

The optimization algorithm ‘relax_rxns’ provided in the supplementary material ‘S1_Dataset’ under the folder named ‘code’ is used to predict the reversal to epithelial phenotype as follows

load(“EGFR_M”); % provided in the supplementary material ‘S1_Dataset’

load(“VE”); % provided in the supplementary material ‘S1_Dataset’

[relaxed_model, ~, d, ~, rxns_relaxed] = relax_rxns(EGFR_M,[],[],[],VE,0.9);

VE is the target flux, i.e the flux of the epithelial model EGFR_E. The reversed model is returned in “relaxed_model”. “rxns_relaxed” contains the five reactions whose flux bounds need to be altered in order to change EGFR_M to EGFR_E. These five reactions are listed in section 2.2.1.

Table S1 shows the genes related to these five reactions.They were identified with *findGenesFromRxns*.

**geneList = findGenesFromRxns(model, reactions)**

**We found 22 unique genes to be related to these five reactions, however, when we manually checked the expression of these 22 genes, we found that** *MAPK1*, *NRAS, HRAS and EGFR* genes were overexpressed in D492 compared to D492M and the inhibitor *PTEN* was overexpressed in D492M. We thus hypothesized that these genes were responsible for the flux differences in these two models.

1. **The effects of active AKT signaling on the EMT metabolic network.**

The connection of the signaling network to the metabolic network was done by predicting flux differences within AKT signaling in EGFR_E and EGFR_M as described in section 11 here and extrapolating from these differences using data from the literature describing the effects of signaling on metabolic gene expression.

The results are shown in Table 1 in the main manuscript. We examined how the metabolic genes which are affected by AKT signaling impact other metabolic pathways by constraining the EMT metabolic network with the metabolic genes known to be affected by AKT signaling. We constrained RECON 2 [10] using the microarray data from Sigurdsson *et al.* [1] to generate an EMT metabolic network (submitted to Biomodels: MODEL1602080000, unpublished). This reconstruction consists of all the metabolic reactions encoded in both the D492 and D492M cell lines. This network has GPRs connecting each reaction with the genes of the enzymes catalyzing the reaction. Through GPRs, we were able to associate metabolic genes affected by AKT signaling to the metabolic reactions in the EMT network by the *findRxnsFromGene*‘ function. Thus, the metabolic genes predicted to be up-regulated in the epithelial cells due to AKT signaling (**Table 1** in the main manuscript) led to the identification of up-regulated reactions in D492 and D492M. This information was then used to regulate the flux bounds of the affected reactions. Specifically, up-regulation in an epithelial metabolic model was simulated by downregulating the corresponding reaction in its mesenchymal counterpart, and vice versa (also described in methods section 4.4 and 4.7 in the main manuscript). Flux bounds of the metabolic reactions up-regulated due to AKT signaling in D492 were constrained by an arbitrary factor, 1/100 of the initial bounds in D492M and vice versa. This led to formation of Met_E and Met_M (epithelial and mesenchymal metabolic networks). The flux values through each reaction in both the models were determined through random sampling and the relative flux span, s_M_(i)/ s_E_(i) was used to quantify the flux differences between the networks. Here, s_M_(i) and s_E_(i) represent the average flux in reaction i for the mesenchymal and epithelial cells, respectively. This way we were able to predict metabolic pathways that may be affected downstream upon activation of AKT signaling in breast epithelial cells D492 and D492M during EMT.

**References**

1. Sigurdsson V, Hilmarsdottir B, Sigmundsdottir H, Fridriksdottir AJR, Ringnér M, Villadsen R, et al. Endothelial induced EMT in breast epithelial cells with stem cell properties. PLoS One. Public Library of Science; 2011;6: e23833. doi:10.1371/journal.pone.0023833

2. Barrett T, Wilhite SE, Ledoux P, Evangelista C, Kim IF, Tomashevsky M, et al. NCBI GEO: archive for functional genomics data sets--update. Nucleic Acids Res. 2013;41: D991–5. doi:10.1093/nar/gks1193

3. Javaid S, Zhang J, Anderssen E, Black JC, Wittner BS, Tajima K, et al. Dynamic Chromatin Modification Sustains Epithelial-Mesenchymal Transition following Inducible Expression of Snail-1. Cell Rep. 2013;5: 1679–1689. doi:10.1016/j.celrep.2013.11.034

4. Tam WL, Lu H, Buikhuisen J, Soh BS, Lim E, Reinhardt F, et al. Protein kinase C α is a central signaling node and therapeutic target for breast cancer stem cells. Cancer Cell. 2013;24: 347–64. doi:10.1016/j.ccr.2013.08.005

5. McGrail DJ, Mezencev R, Kieu QMN, McDonald JF, Dawson MR. SNAIL-induced epithelial-to-mesenchymal transition produces concerted biophysical changes from altered cytoskeletal gene expression. FASEB J. 2015;29: 1280–9. doi:10.1096/fj.14-257345

6. Cai J, Guan H, Fang L, Yang Y, Zhu X, Yuan J, et al. MicroRNA-374a activates Wnt/β-catenin signaling to promote breast cancer metastasis. J Clin Invest. 2013;123: 566–79. doi:10.1172/JCI65871

7. Deshiere A, Duchemin-Pelletier E, Spreux E, Ciais D, Combes F, Vandenbrouck Y, et al. Unbalanced expression of CK2 kinase subunits is sufficient to drive epithelial-to-mesenchymal transition by Snail1 induction. Oncogene. Macmillan Publishers Limited; 2013;32: 1373–83. doi:10.1038/onc.2012.165

8. Schellenberger J, Palsson BØ. Use of randomized sampling for analysis of metabolic networks. J Biol Chem. 2009;284: 5457–61. doi:10.1074/jbc.R800048200

9. Schellenberger J, Que R, Fleming RMT, Thiele I, Orth JD, Feist AM, et al. Quantitative prediction of cellular metabolism with constraint-based models: the COBRA Toolbox v2.0. Nat Protoc. 2011;6: 1290–307. doi:10.1038/nprot.2011.308

10. Thiele I, Swainston N, Fleming RMT, Hoppe A, Sahoo S, Aurich MK, et al. A community-driven global reconstruction of human metabolism. Nat Biotechnol. Nature Publishing Group, a division of Macmillan Publishers Limited. All Rights Reserved.; 2013;31: 419–25. doi:10.1038/nbt.2488
