# Supplementary figures and images for: EGFR Signal-Network Reconstruction Demonstrates Metabolic Crosstalk in EMT
Source: PLoS Comput Biol. 2016 Jun 2;12(6):e1004924. doi: 10.1371/journal.pcbi.1004924 (PMC4890760; doi:10.1371/journal.pcbi.1004924)

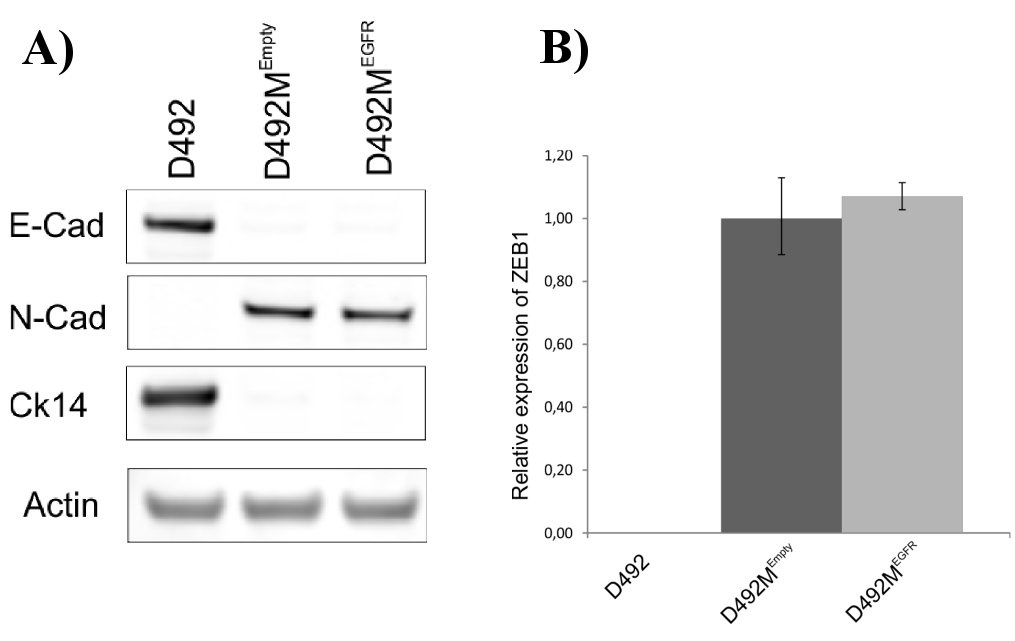

Supplement: S1 Fig — (A) Western blotting for epithelial markers E-Cadherin and CK14 and mesenchymal marker N-Cadherin. Overexpression of EGFR in D492M does not revert the mesenchymal phenotype towards an epithelial phenotype. D492MEGFR retains N-Cadherin expression and does not gain E-Cadherin or CK14 expression. (B) Real-Time Quantitative Reverse Transcription PCR of the EMT transcription factor ZEB1 in D492, D492MEGFR and D492MEmpty normalized to GAPDH. ZEB1 transcription was not detected in D492 and the transcription level of ZEB1 was unchanged in D492MEGFR compared to D492MEmpty. D492MEGFR retains mesenchymal ZEB1 expression. (TIFF) [file pcbi.1004924.s008.tiff]
