# Supplementary material for: EGFR Signal-Network Reconstruction Demonstrates Metabolic Crosstalk in EMT
Source: PLoS Comput Biol. 2016 Jun 2;12(6):e1004924. doi: 10.1371/journal.pcbi.1004924 (PMC4890760; doi:10.1371/journal.pcbi.1004924)
Supplement: S1 Table — (DOCX) [file pcbi.1004924.s009.docx]

| **Entry** | **Protein names** | **Gene names** |
| --- | --- | --- |
| P28482 | Mitogen-activated protein kinase 1 (MAP kinase 1) | MAPK1 |
| P27361 | Mitogen-activated protein kinase 3 (MAP kinase 3) | MAPK3 |
| Q02750 | Dual specificity mitogen-activated protein kinase kinase 1 (MAP kinase kinase 1) | MAP2K1 |
| P36507 | Dual specificity mitogen-activated protein kinase kinase 2 (MAP kinase kinase 2) | MAP2K2 |
| P31946 | 14-3-3 protein beta/alpha (Protein 1054) (Protein kinase C inhibitor protein 1) | YWHAB |
| Q8IVT5 | Kinase suppressor of Ras 1 | KSR1 |
| P27448 | MAP/microtubule affinity-regulating kinase 3 (EC 2.7.11.1) (C-TAK1) (cTAK1) (Cdc25C-associated protein kinase 1) (ELKL motif kinase 2) (EMK-2) (Protein kinase STK10) (Ser/Thr protein kinase PAR-1) (Par-1a) (Serine/threonine-protein kinase p78) | MARK3 |
| P01112 | GTPase HRas (H-Ras-1) (Ha-Ras) | HRAS |
| P01111 | GTPase NRas | NRAS |
| P01116 | GTPase Kras | KRAS |
| P15056 | Serine/threonine-protein kinase B-raf | BRAF |
| P04049 | RAF proto-oncogene serine/threonine-protein kinase | RAF1 |
| Q16828 | Dual specificity protein phosphatase 6 | DUSP6 |
| Q16829 | Dual specificity protein phosphatase 7 | DUSP7 |
| Q99956 | Dual specificity protein phosphatase 9 | DUSP9 |
| P00533 | Epidermal growth factor receptor | EGFR |
| P27986 | Phosphatidylinositol 3-kinase regulatory subunit alpha | PIK3R1 |
| P62993 | Growth factor receptor-bound protein 2 | GRB2 |
| P42336 | Phosphatidylinositol 4,5-bisphosphate 3-kinase catalytic subunit alpha isoform | PIK3CA |
| P01133 | Pro-epidermal growth factor (EGF) | EGF |
| Q13480 | GRB2-associated-binding protein 1 (GRB2-associated binder 1) | GAB1 |
| P60484 | Phosphatidylinositol 3,4,5-trisphosphate 3-phosphatase and dual-specificity protein phosphatase PTEN | PTEN |

***Table S1: Gene associated with the reactions important for the reversal of EGFR_M to EGFR_E.***
