# Supplementary material for: EGFR Signal-Network Reconstruction Demonstrates Metabolic Crosstalk in EMT
Source: PLoS Comput Biol. 2016 Jun 2;12(6):e1004924. doi: 10.1371/journal.pcbi.1004924 (PMC4890760; doi:10.1371/journal.pcbi.1004924)
Supplement: S4 Table — ER: Microarray Expression data in TWIST, SLUG and SNAIL induced HMLE cells respectively. PE: Proposed Expression. Predictions in agreement with microarray data are highlighted in green and that otherwise are highlighted in orange. (DOCX) [file pcbi.1004924.s012.docx]

| **Metabolic genes** | **PE** | **MCF-7_mirna** | **MCF-7_snail** |
| --- | --- | --- | --- |
| ACC (acetyl-CoA carboxylase alpha) | ↓ E | ↓ M | ↓ E |
| ACLY( ATP-citrate synthase) | ↓ E | p-value insignificant | ↓ E |
| ATIC (Bifunctional purine biosynthesis protein PURH) | ↓ E | ↓ M | p-value insignificant |
| FASN (Fatty acid synthase) | ↓ E | ↓ E | p-value insignificant |
| GAPDH (Glyceraldehyde-3-phosphate dehydrogenase) | ↓ E | ↓ E | p-value insignificant |
| GLUT1 (facilitated glucose transporter) | ↓ E | ↓ E | ↓ M |
| GYS1 (Glycogen [starch] synthase, muscle) | ↓ E | ↓ E | p-value insignificant |
| HK1 (Hexokinase-1) | ↓ E | ↓ E | ↓ M |
| HK2 (Hexokinase-2) | ↓ E | p-value insignificant | ↓ E |
| HMGCR (3-hydroxy-3-methylglutaryl coenzyme A reductase) | ↓ E | ↓ E | p-value insignificant |
| HMGCS1 (Hydroxymethylglutaryl-CoA synthase, cytoplasmic) | ↓ E | ↓ E | ↓ E |
| HPRT1 (Hypoxanthine-guanine phosphoribosyltransferase) | ↓ E | p-value insignificant | ↓ E |
| ME1 (Malic enzyme) | ↓ E | p-value insignificant | ↓ E |
| PFKFB2 (6-phosphofructo-2-kinase/fructose-2,6-bisphosphatase 2) | ↓ E | ↓ E | ↓ M |
| SREBF1 (Sterol regulatory element-binding protein 1) | ↓ E | ↓ E | ↓ M |
| SREBF2 (Sterol regulatory element-binding protein 2) | ↓ E | ↓ E | p-value insignificant |
| TALDO1 (Transaldolase) | ↓ E | p-value insignificant | ↓ E |
| TKT (Transketolase) | ↓ E | ↓ E | ↓ E |
| G6PC (Glucose-6-phosphatase) | ↓ M | p-value insignificant | ↓ M |
| PCK1 (Phosphoenolpyruvate carboxykinase 1) | ↓ M | ↓ M | p-value insignificant |

***Table S4: Predicted expression of metabolic genes regulated by AKT in MCF7 cells.*** *ER: Microarray Expression data in TWIST, SLUG and SNAIL induced HMLE cells respectively. PE: Proposed Expression.* *Predictions in agreement with microarray data are highlighted in green and that otherwise are highlighted in orange*
