# Supplementary material for: EGFR Signal-Network Reconstruction Demonstrates Metabolic Crosstalk in EMT
Source: PLoS Comput Biol. 2016 Jun 2;12(6):e1004924. doi: 10.1371/journal.pcbi.1004924 (PMC4890760; doi:10.1371/journal.pcbi.1004924)
Supplement: S5 Table — “Reference” column lists the studies from which the influence of AKT signaling on the expression of the corresponding metabolic genes was derived. +1 and -1 denotes positive and negative regulation, respectively. (DOCX) [file pcbi.1004924.s013.docx]

| **References** | **Metabolic Genes** | **Regulation** |
| --- | --- | --- |
| [1] | *GAPDH (Glyceraldehyde-3-phosphate dehydrogenase)* | +1 |
| [2] | *GLUT1 (facilitated glucose transporter)* | +1 |
| [3,4] | *GYS1 (Glycogen [starch] synthase, muscle)* | +1 |
| [5,6] | *HK1 (Hexokinase-1)* | +1 |
| [5,6] | *HK2 (Hexokinase-2)* | +1 |
| [4] | *G6PC (Glucose-6-phosphatase)* | -1 |
| [4] | *PCK1 (Phosphoenolpyruvate carboxykinase 1)* | -1 |
| [3] | *ACLY( ATP-citrate synthase)* | +1 |
| [3] | *ME1 (Malic enzyme)* | +1 |
| [7,8] | *PFKFB2 (6-phosphofructo-2-kinase/fructose-2,6-bisphosphatase 2)* | +1 |
| [3] | *HMGCR (3-hydroxy-3-methylglutaryl coenzyme A reductase)* | +1 |
| [3] | *HMGCS1 (Hydroxymethylglutaryl-CoA synthase, cytoplasmic)* | +1 |
| [9] | *ACC (acetyl-CoA carboxylase alpha)* | +1 |
| [9] | *SREBF1 (Sterol regulatory element-binding protein 1)* | +1 |
| [3,9] | *SREBF2 (Sterol regulatory element-binding protein 2)* | +1 |
| [9] | *FASN (Fatty acid synthase)* | +1 |
| [10] | *ATIC (Bifunctional purine biosynthesis protein PURH)* | +1 |
| [10] | *HPRT1 (Hypoxanthine-guanine phosphoribosyltransferase)* | +1 |
| [10] | *TALDO1 (Transaldolase)* | +1 |
| [11] | *TKT (Transketolase)* | +1 |

***Table S5: Regulation of metabolic gene expression by AKT signaling.*** *“Reference” column* *lists the studies from which the influence of AKT signaling on the expression of the corresponding metabolic genes was derived. +1 and -1 denotes positive and negative regulation, respectively.*

**References:**

1. Shen W. Akt and Mammalian Target of Rapamycin Regulate Separate Systems of Proteolysis in Renal Tubular Cells. J Am Soc Nephrol. 2006;17: 2414–2423. doi:10.1681/ASN.2005111157

2. Barthel A, Okino ST, Liao J, Nakatani K, Li J, Whitlock JP, et al. Regulation of GLUT1 Gene Transcription by the Serine/Threonine Kinase Akt1. J Biol Chem. 1999;274: 20281–20286. doi:10.1074/jbc.274.29.20281

3. Porstmann T, Griffiths B, Chung Y-L, Delpuech O, Griffiths JR, Downward J, et al. PKB/Akt induces transcription of enzymes involved in cholesterol and fatty acid biosynthesis via activation of SREBP. Oncogene. 2005; 6465–6481. doi:10.1038/sj.onc.1208802

4. Lochhead PA, Coghlan M, Rice SQ, Sutherland C. Inhibition of GSK-3 selectively reduces glucose-6-phosphatase and phosphatase and phosphoenolypyruvate carboxykinase gene expression. Diabetes. 2001;50: 937–46. Available: http://www.ncbi.nlm.nih.gov/pubmed/11334436

5. Miyamoto S, Murphy AN, Brown JH. Akt mediates mitochondrial protection in cardiomyocytes through phosphorylation of mitochondrial hexokinase-II. Cell Death Differ. 2008;15: 521–9. doi:10.1038/sj.cdd.4402285

6. Vander Heiden MG, Plas DR, Rathmell JC, Fox CJ, Harris MH, Thompson CB. Growth factors can influence cell growth and survival through effects on glucose metabolism. Mol Cell Biol. 2001;21: 5899–912. Available: http://www.pubmedcentral.nih.gov/articlerender.fcgi?artid=87309&tool=pmcentrez&rendertype=abstract

7. Novellasdemunt L, Tato I, Navarro-Sabate A, Ruiz-Meana M, Méndez-Lucas A, Perales JC, et al. Akt-dependent activation of the heart 6-phosphofructo-2-kinase/fructose-2,6-bisphosphatase (PFKFB2) isoenzyme by amino acids. J Biol Chem. 2013;288: 10640–51. doi:10.1074/jbc.M113.455998

8. Elstrom RL, Bauer DE, Buzzai M, Karnauskas R, Harris MH, Plas DR, et al. Akt stimulates aerobic glycolysis in cancer cells. Cancer Res. 2004;64: 3892–9. doi:10.1158/0008-5472.CAN-03-2904

9. Yoon S, Lee MY, Park SW, Moon JS, Koh YK, Ahn YH, et al. Up-regulation of acetyl-CoA carboxylase alpha and fatty acid synthase by human epidermal growth factor receptor 2 at the translational level in breast cancer cells. J Biol Chem. 2007;282: 26122–26131. doi:10.1074/jbc.M702854200

10. Wang W, Fridman a., Blackledge W, Connelly S, Wilson I a., Pilz RB, et al. The Phosphatidylinositol 3-Kinase/Akt Cassette Regulates Purine Nucleotide Synthesis. J Biol Chem. 2009;284: 3521–3528. doi:10.1074/jbc.M806707200

11. Arindam Saha, Stephen Connelly, Jingjing Jiang, Shunhui Zhuang DTA, Tony Phan, Renate B. Pilz and GRB. Akt Phosphorylation and Regulation of Transketolase Is a Nodal Point for Amino Acid Control of Purine Synthesis. Mol Cell. 2014;55: 264–276. doi:10.1016/j.micinf.2011.07.011.Innate
