# Supplementary material for: EGFR Signal-Network Reconstruction Demonstrates Metabolic Crosstalk in EMT
Source: PLoS Comput Biol. 2016 Jun 2;12(6):e1004924. doi: 10.1371/journal.pcbi.1004924 (PMC4890760; doi:10.1371/journal.pcbi.1004924)
Supplement: S1 Appendix — (DOCX) [file pcbi.1004924.s014.docx]

**Appendix S1**

**Full form of abbreviations**

AKT v-akt murine thymoma viral oncogene homolog 1

ErBb2 erb-b2 receptor tyrosine kinase 2

MAPK Mitogene-activated kinase-like protein

CaM calmodulin

IP3 inositol 1,4,5-trisphosphate

HER2 human epidermal growth factor receptor 2
